# Host fecal DNA specific methylation signatures mark gut dysbiosis and inflammation in children affected by autism spectrum disorder

**DOI:** 10.1038/s41598-023-45132-0

**Published:** 2023-10-24

**Authors:** Mariella Cuomo, Lorena Coretti, Davide Costabile, Rosa Della Monica, Giulia De Riso, Michela Buonaiuto, Federica Trio, Carmela Bravaccio, Roberta Visconti, Roberto Berni Canani, Lorenzo Chiariotti, Francesca Lembo

**Affiliations:** 1 Department of Molecular Medicine and Medical Biotechnologies, University of Naples “Federico II”, Via S. Pansini 5, 80131 Naples, Italy; 2 Present Address: CEINGE Advanced Biotechnologies “Franco Salvatore”, Via G. Salvatore 482, 80145 Naples, Italy; 3 Department of Pharmacy, University of Naples “Federico II”, Via Domenico Montesano 49, 80131 Naples, Italy; 4 SEMM-European School of Molecular Medicine, University of Naples “Federico II”, Naples, Italy; 5 Department of Translational Medical Science - Pediatric Section, University of Naples Federico II, Naples, Italy; 6 Institute for the Experimental Endocrinology and Oncology “G. Salvatore”, Italian National Council of Research, Via S. Pansini 5, 80131 Naples, Italy

**Keywords:** Microbiology, Molecular medicine

## Abstract

The gut-brain axis involves several bidirectional pathway communications including microbiome, bacterial metabolites, neurotransmitters as well as immune system and is perturbed both in brain and in gastrointestinal disorders. Consistently, microbiota-gut-brain axis has been found altered in autism spectrum disorder (ASD). We reasoned that such alterations occurring in ASD may impact both on methylation signatures of human host fecal DNA (HFD) and possibly on the types of human cells shed in the stools from intestinal tract giving origin to HFD. To test this hypothesis, we have performed whole genome methylation analysis of HFD from an age-restricted cohort of young children with ASD (N = 8) and healthy controls (N = 7). In the same cohort we have previously investigated the fecal microbiota composition and here we refined such analysis and searched for eventual associations with data derived from HFD methylome analysis. Our results showed that specific epigenetic signatures in human fecal DNA, especially at genes related to inflammation, associated with the disease. By applying methylation-based deconvolution algorithm, we found that the HFD derived mainly from immune cells and the relative abundance of those differed between patients and controls. Consistently, most of differentially methylated regions fitted with genes involved in inflammatory response. Interestingly, using Horvath epigenetic clock, we found that ASD affected children showed both epigenetic and microbiota age accelerated. We believe that the present unprecedented approach may be useful for the identification of the ASD associated HFD epigenetic signatures and may be potentially extended to other brain disorders and intestinal inflammatory diseases.

## Introduction

It is now well established that a direct and indirect connection between gut and brain exists and several pathways have been identified as involved in the communication between the two organs^1,2^. Some of these pathways employs the gut microbiota metabolites, in particular the Short Chain Fatty Acids (SCFAs) that may exert their effect in various ways, including the generation of epigenetic changes^3^. Therefore, alterations in gut microbiota composition may likely be mirrored by chromatin and/or DNA methylation changes^4,5^. This phenomenon may affect many diseases in which it has been demonstrated abnormal gut microbiota composition, including Autism Spectrum Disorders (ASD)^6–8^. Despite ASD remain a disease with a still unclear etiology, the presence of both a neuroinflammatory component and widespread intestinal inflammation, with the presence of the so-called “leaky gut”, has been widely demonstrated^7,9–12^. Additionally, the involvement of aberrant DNA methylation in the etiopathogenesis of ASD has become increasingly evident at multiple levels, from genetic mutations in epigenetic readers (for example, at MeCP2 gene^13^) to gene-specific and genome-wide epigenetic changes^14,15^. The majority of these latter studies aimed to detect specific DNA methylation marks in post-mortem brain tissues^16,17^ to dissect the epigenetic clues causing the disease, or in peripheral blood^18–20^, to search for "episignatures" that may be easily used as biomarkers of the disease. However, if and how an unbalance in gut microbiota composition may directly elicit its effect on the epigenome is still under investigation. To this aim, the study of colon epithelial cell lines exposed to specific microbes suggests a direct relationship between gut microbes, gene methylation, and the development of diseases^21^. As an alternative in vivo method, the use of the human fecal DNA and RNA may be representative of exfoliant epithelial cells and, in some cases, inflammatory intestinal cells, allowing the evaluation of cells that have been in direct contact with gut microbiota metabolites^22,23^. Notably, recent studies have demonstrated that the host transcriptomic analysis of fecal washes is a sensitive predictor of intestinal inflammation not only by identifying the changes in the expression levels of inflammatory-related genes but also by deconvoluting the percentage of different types of immune cells^24,25^.

To our knowledge, no studies have investigated the possibility to identify epigenetic changes in human fecal DNA in diseases showing gut microbiota alteration, such as the ASD. We have previously demonstrated that children, in a very tight range of age (from 2 to 4 years old) and at first diagnosis of ASD, presented an altered microbiota composition, including abnormalities in temporal colonization of specific bacterial strains, together with higher fecal levels of butyric acid^8^. Here, we report the epigenome analyses on fecal DNA in children suffering ASD, previously characterized for gut microbiota, to explore the DNA methylation signatures in HFD likely resembling the epithelial and immune intestinal cells, and so, cells that have been in close contact with gut microbiota ecosystem. We performed genome-wide as well as region-specific DNA methylation analyses in HFD from 8 children affected by ASD (ASD group) and 7 non-affected children (CTRL group). We found several differentially methylated CpG sites and regions, the majority of those belonging to inflammatory pathways and lipid metabolism pathways. Furthermore, the changes in DNA methylation were ascribed to an increase in pro-inflammatory cells, in particular neutrophils, in stools of ASD affected children. Finally, we found that ASD affected children presented a statistically significant accelerated “epigenetic age” that well correlates with their “microbiota age”, in terms of increased levels of some bacterial species commonly found in the adultness.

## Results

### Methylome differences in fecal DNA of ASD children compared to non-affected children

**Figure 1:**
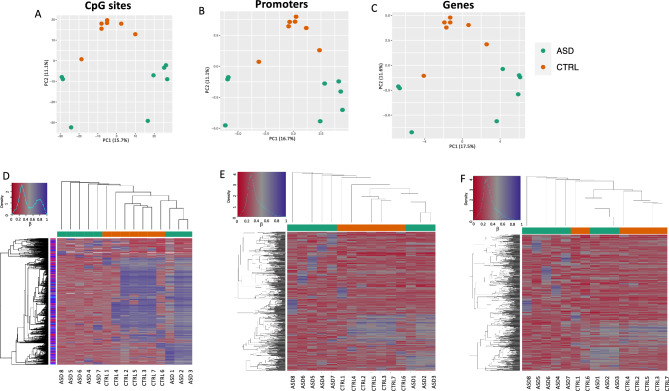
Methylome analyses of ASD and CTRL groups. Principal Component Analysis plots of DNA methylation profiles showing cluster of sample groups (in orange, CTRL group (N = 7); in green ASD group (N = 8)) based on the quality filtered CpG sites (**A**), promoters (**B**) and genes (**C**). Hierarchical cluster analyses based on DNA methylation levels at 1000 most variable CpG sites (**D**), promoters (**E**) and genes (**F**).

In our previous study^8^, we demonstrated striking differences in gut microbiota composition in first-diagnosed young children affected by ASD compared to controls. Refinement of such analysis (see Supplementary Fig. S1 and Supplementary Methods) confirmed that the fecal microbial community in ASD children was enriched in species potentially stimulating pro-inflammatory response. Here, we wondered whether, in the same cohort of children, we could be able to identify disease-related epigenome signatures in HFD. To this aim, we performed 850 k Epic Array methylome analysis on HFD from 8 children affected by ASD and 7 non-affected children (32.75 ± 4.02 and 32.87 ± 6.99 months of age, respectively). We started by analyzing the DNA methylation profiles considering all the quality-filtered array probes (Fig. 1). To do this, we performed principal component analysis (PCA) by clustering samples according to the level of methylation at single CpG sites, genes and promoters (Fig. 1A–C). Both PC1 and PC2 separated the two sample groups with a variance explained of more than 25%. The computed sample coordinates in the principal component space were then tested for association with the sample groups. We found that the second component significantly correlated with the disease status (CpG site *p* = *0.0003*; Promoters *p* = *0.001*; Genes *p* = *0.003*). We then performed hierarchical clustering based on the methylation levels at sites/regions with the highest variance across all samples (Fig. 1D–F). Also in doing so, we found that, especially at CpG sites and promoter levels, a hierarchical cluster of healthy controls (in orange) and 5/8 of ASD-affected samples (in green) was present. Thus, at epigenome-wide levels, in HFD, children at first diagnosis of ASD were distinguishable from non-affected children.

### DNA methylation changes in ASD children at inflammatory pathways

**Figure 2:**
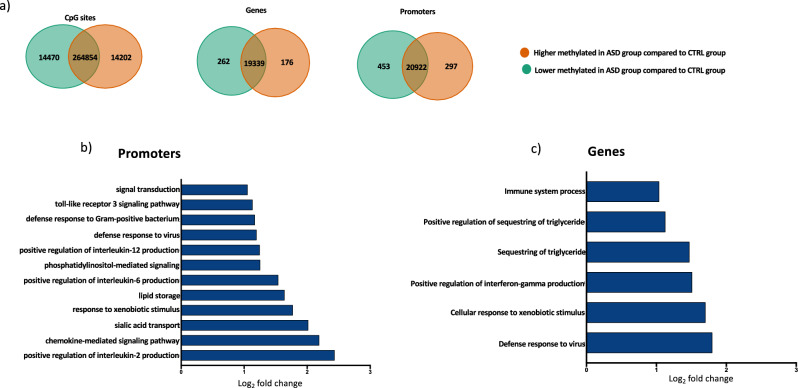
Differentially methylated loci between ASD and CTRL groups and Gene ontology analysis. (**a**) Venn diagrams showing the CpG sites, genes and promoters significantly lower methylated (in green, *p* value < 0.05) in ASD group compared to CTRL group and the CpG sites, genes and promoters significantly higher methylated (in orange, *p* value < 0.05) in ASD group compared to CTRL group. The intersect of Venn Diagrams indicate CpG sites, promoters and genes that were no differentially methylated between the two groups. Bar charts showing the top GO terms for biological process, considering the promoters (**b**) and genes (**c**) significantly lower methylated in the ASD group (CTRL group, N = 7; ASD group, N = 8).

Our next aim was to determine the DNA methylation degree at higher resolution, considering the significant differentially methylated CpG sites and specific regions (Fig. 2a). We identify 28,672 differentially methylated CpG sites (*p* < *0.05*), 483 differentially methylated genes (*p* < *0.05*) and 750 differentially methylated promoters. Among the CpG sites, similar number of CpG sites were hypomethylated or hypermethylated in ASD samples (14,470 hypomethylated in ASD and 14,202 hypermethylated in ASD), while the majority of differentially methylated genes and promoters were essentially hypomethylated in the ASD group (Fig. 2a). Thus, methylome analysis from HFD allowed the discrimination of the ASD group from the healthy controls at epigenome-wide level as well as the identification of several differentially methylated sites and regions characterizing the disease. To evaluate the biological roles of differentially methylated regions, we performed gene ontology on hypo- and hypermethylated genes and promoters in the ASD group. Differentially methylated regions were analyzed by evaluating the enriched biological processes and separating the hypo- and hypermethylated genes and promoters (Fig. 2b,c). Due to the lower number of hypermethylated promoters and genes in ASD (Fig. 2a), no significant enriched pathways were found when analyzing the hypermethylated regions. We then examined the hypomethylated promoters (Fig. 2b). We found a strong enrichment of inflammatory and immune pathways, such as “toll-like receptor 3 signaling pathway”, “defense response to Gram-positive bacteria”, “defense response to virus”, “positive regulation of interleukin-12 production”, “positive regulation of interleukin-6 production”, “chemokine-mediated signaling pathway” and “positive regulation of interleukin-2 production”. We also identified some pathways involved in the lipid metabolism, such as “phosphatidylinositol-mediated signaling” and “lipid storage”. Similar results were found also for the hypomethylated genes (Fig. 2c). Indeed, we found enrichment of the pathways “Immune system process”, “Positive regulation of interferon-gamma production” and “Defense response to virus”, together with pathways involved in the lipid metabolism such as “Positive regulation of sequestering of triglyceride”. Thus, the majority of the hypomethylated genes and promoters in the ASD group were collocated in inflammatory and immune pathways. All the significant differentially methylated promoters are shown in Supplementary Table S1 and Supplementary Table S2. Among the inflammatory and immune-related hypomethylated promoters, we found IL-6 and IL-1B promoters significantly hypomethylated in children affected by ASD. Both interleukins are involved in pro-inflammatory response as they act in response to specific bacterial antigens, such as LPS^26,27^. Thus, the lower levels of DNA methylation in ASD children may likely correspond to an increase of their expression associated with an enhanced inflammatory status. Interestingly, also toll like receptor 3 (TLR3) promoter was found hypomethylated in the ASD group. This gene is mainly involved in antiviral immune response^28^ and it is also able to initiates a series of signals that trigger the production of interferon regulatory factor 3 and other cytokines, including IL-6, ultimately stimulating the immune and inflammatory response^29^. Moreover, also promoters of some chemokine and chemokine receptors, such as CXCL13 and CXCR3, showed a significant lower degree of DNA methylation in the stool of children affected by ASD. Also in this case, the hypomethylation of these proinflammatory cytokines may correlate with the increased colon inflammation in the ASD group. In addition to the above-described cytokines, we also found hypomethylation at promoters of genes involved in lipid storage, such as the diacylglycerol O-acyltransferase 1 (DGAT1) gene. The protein codified by the DGAT1 gene has the capability to metabolize the fatty acyl-CoA to triacylglycerol and is highly expressed in small intestine^30^. Mutations in DGAT1 have been related to chronic and severe diarrhea, especially during the neonatal period^31,32^. Since children affected by ASD often suffer of chronic diarrhea^33^, the hypomethylation in DGAT1 gene in the ASD group may likely be related to this clinical feature of ASD. Despite it remains to be clarified whether these changes in DNA methylation may be a cause or a consequence of gut microbiota alteration, our data indicate that HFD methylation in ASD children changes globally and at specific genes, especially those involved in inflammation and immunity.

### Immune cell type identification from HFD methylome of CTRL and ASD groups

**Figure 3:**
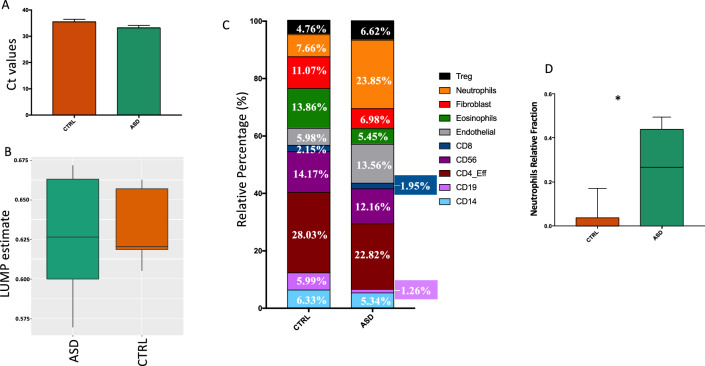
Cell type deconvolution from fecal DNA. (**A**) Ct values of GAPDH amplification in order to quantify HFD in CTRL and ASD groups. (**B**) Immune cell amount estimation in CTRL and ASD samples by applying the LUMP algorithm. (**C**) Stacked bar plots showing the relative percentage of specific cell-type in CTRL and ASD groups based on the MethylCIBERSORT algorithm. (**D**) Neutrophils relative percentage in CTRL and ASD groups. (*p value = 0.03). (CTRL group, N = 7; ASD group, N = 8).

The above-mentioned gene ontology analyses on HFD showed an enrichment of immune-related pathways. These differences may be either due to cellular dynamic DNA methylation changes between healthy controls and patients affected by ASD or, alternatively, to different cell type composition generating the HFD content. By applying a reference-based algorithm for the inference of cell-type proportions on HFD, we could estimate the relative percentage of endothelial cells, fibroblasts, and total immune cells in the samples (Fig. 3). First of all, in order to quantify the amount of HFD in each sample, we amplified the human GAPDH house-keeping gene on DNA extracted from 150 mg of stools from each sample in both groups. We did not observe differences in the percentage of human DNA in stools of ASD and CTRL groups indicating that in both groups the number of cells shedding in the colonic lumen was similar (Fig. 3A). Then, we applied the Leukocytes Unmethylation for Purity (LUMP) (Fig. 3B)^34^, identifying the percentage of immune cells in the analyzed samples. We found an average of 63% of immune cells both in ASD and CTRL group, with a higher variability in ASD children. Then, we tried to depict more accurately the cell type composition in the two groups by applying the computational approach MethylCIBERSORT (Fig. 3C)^35,36^. By using this algorithm, we were able to quantify the portion of Treg cells, Neutrophils, Fibroblasts, Eosinophils, Endothelial cells, CD8, CD56, CD4, CD19 and CD14 cell types (Fig. 3C). However, this algorithm does not identify the percentage of epithelial cells. We found different patterns but an almost similar distribution of all type of cells with exception of neutrophils. In fact, we found that about 30% of the cells belonging to ASD samples were neutrophils while the amount of these cells was very low in the CTRL group, with statistically significant differences between the two groups (Fig. 3D). The presence of neutrophils into the colon mucosa is considered a hallmark of active and severe inflammation^37^. To our knowledge, no cell-type deconvolution studies have been conducted on HFD. Thus, despite we were not able to state the effective cellular composition from healthy control fecal samples, deconvolution analysis allowed us to identify differences in immune cell percentage between the healthy and the ASD groups.

### Epigenetic age in children affected by ASD is higher compared to non-affected children

**Figure 4:**
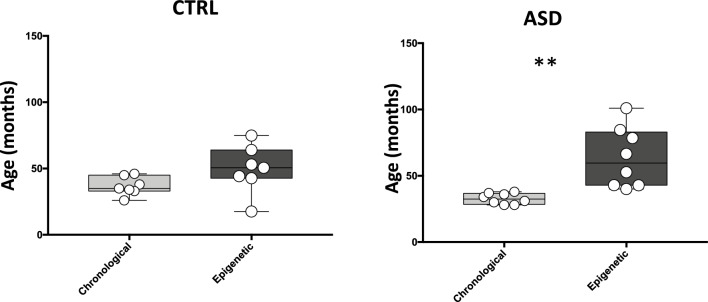
Chronological and Epigenetic Ages. Box Plots showing the chronological and epigenetic age in CTRL (N = 7) and ASD (N = 8) groups. Age is showed as months. (**p value < 0.01).

In our previous study^8^, we found that the gut microbiota of children at first diagnosis of ASD presented a depletion of key bacterial species typical of infant gut microbiota and an increase of bacterial species mainly found in adult gut microbiota. Thus, from a microbiota point of view, the age of ASD children was likely higher compared to their chronological age. Here, we decided to calculate the epigenetic age by utilizing the Horvath algorithm^38^ on the methylome data obtained from HFD (Fig. 4). We found a significant increase (*p* < *0.05*) in epigenetic age in ASD children, with a chronologic age average of 32.75 ± 1.42 months and an epigenetic age that duplicated in months (63.64 ± 8.02 months). Conversely, no significant differences were found in healthy controls (Fig. 4). Investigation of the relationship between epigenetic age and the relative abundance of discriminant bacterial taxa between the two groups showed a remarkable negative association of *Streptococcus* with epigenetic age acceleration, while a positive association was found in the case of Ruminococcaceae (Supplementary Fig. S1D). Other near-significant positive associations with age acceleration were observed in the case of *Bacteroides vulgatus* and two Firmicutes species, the mucin-degrader *Ruminococcus torques* and *Eubacterium halii*.

Overall, we found an acceleration of epigenetic age associated with an adult-type gut ecosystem in the here analyzed young ASD patients, suggesting a premature biological ageing of ASD children. To our knowledge, this is the first study evaluating the epigenetic clock on HFD and that explores the potential applicability of epigenetic clock in ASD in tissues different from blood.

## Discussion

Host fecal DNA is emerging as a powerful method for non-invasive, nucleic acid-based detection of GI diseases^25,39,40^. Especially in colorectal cancer, host fecal DNA is currently used for diagnosis, screening and evaluation of progression of the disease, since cancerous colonic epithelial cells continuously exfoliate^41,42^. In other non-neoplastic gastrointestinal diseases, the analysis of HFD amount along with fecal RNA transcriptome analysis has been proposed as a tool to search for specific signatures of gut inflammation and leaky gut condition^25,43^. However, HFD epigenomic profiles have been investigated only in intestinal neoplastic diseases^44,45^. In the present study, we analyzed for the first time the DNA methylation profiles of host fecal DNA in young children at first diagnosis of ASD, a cohort that we have previously characterized for gut microbiota composition^8^. Despite the small size of the analyzed cohort, we found significant methylation differences at epigenome level (Fig. 1) as well as at specific regions, such as genes and promoters (Fig. 2). The observed phenomena are in line with the results of others’ previous studies demonstrating that transcriptomic analyses of host fecal RNA may predict the presence of intestinal inflammation and, interestingly, some key genes, such as IL1B, found over-expressed in this study^43^ were found here hypomethylated (Supplementary Table S1).

The striking observation that epigenome profiles of ASD patient- and healthy control-derived HFD clustered sharply in a PCA (Fig. 1a–c) prompted us to address the question whether these changes were mainly due to gross changes in fecal cell-type content or to dynamic DNA methylation changes in conserved cell populations. Our results of deconvolution analysis of HFD methylome showed that cellular composition indeed changed, being neutrophils much more represented in ASD stools (Fig. 3). However, the limitation of the current tool to analyze cellular content by deconvoluting methylome data did not allow us to determine, for example, the number and type of exfoliant epithelial cells. In fact, the majority of DNA methylation deconvolution-based classifiers work better on blood cells and tumor microenvironment^46,47^. Another limitation of this study is that we cannot conclude that all the methylation changes we observed are due to cell type changes, instead it remains possible that a not quantifiable quote of changes was due to dynamic changes of methylation patterns in cells possibly due to gut microbiota alteration and/or gut inflammatory state and leaky gut.

Finally, we here calculated the epigenetic age of patients and controls by the analysis of HFD methylomes. Epigenetic age is a recently established biomarker of aging^48^. DNA methylation-based aging clocks is a composite measure of DNA methylation (DNAm) levels across specific CpG sites along the genome and may be calculated from methylome raw data using different computational tools^38,49–51^. Epigenetic age may be largely influenced by several genetic and environmental factors^52,53^ including, possibly, gut microbiota composition and disease-related microbiota variations. By this view, our data, along with metataxonomic analysis results, showed a clear acceleration of epigenetic age in ASD children that correlates with the alteration in abundance of specific colonizers marking the transition from an infant- to adult-like gut microbiota. These findings coming from HFD methylome raw data are particularly intriguing and surely deserve further extensive investigation.

In summary, the striking differences in the HFD methylation profiles here observed and the enrichment of differentially methylated regions at inflammatory genes and promoter, strongly suggest that, upon future appropriate enlargement and extension, the analysis of HFD methylation pattern is a very promising approach to find novel, easy-to-use, biological markers for different human diseases that are associated with gut dysbiosis, inflammatory state of intestine and leaky gut features.

## Conclusions

To date, HFD epigenomic profiles have been investigated only in intestinal neoplastic diseases. In this study, we have demonstrated that HFD methylome analysis can be also used to discriminate children with ASD respect to healthy individuals. In detail, methylome analysis identified in children with ASD a strong enrichment for hypomethylated genes and promoters collocated in inflammatory and immune pathways. Notably, by applying a reference-based algorithm for the inference of cell-type proportions on HFD, we have showed a specific increase in neutrophils generating the HFD content in children with ASD, in accordance with the presence of a severe and active inflammation of the colon mucosa. Finally, by utilizing the Horvath algorithm, the methylome data obtained from HFD showed a significant increase in epigenetic age in children with ASD. Overall, our data show that the analysis of HFD methylation pattern is a very promising approach to find novel biomarkers for ASD, suggesting, moreover, that it could be useful also for characterizing other human diseases affecting gut.

## Methods

### Study subjects

All participants were selected from a previous investigation that aimed to identify the key features of gut microbiota profile and to quantify the fecal SCFAs levels in very young children (2–4 years of age) at first diagnosis of ASD with respect to age-matched neurotypical healthy controls^8^. In the present study we investigated methylome differences in fecal DNA collected from 8 children at first diagnosis of ASD (average age 32.75 ± 4.02 months) and 7 age-matched healthy subjects as controls (average age 32.87 ± 6.99 months). Based on neuropsychiatric assessment, according to the Diagnostic and Statistical Manual of Mental Disorders, Fifth edition (DSM-5)^54^ severity-levels, 4 patients obtained scores indicating the necessity of a very substantial support and 4 the necessity of a substantial support to manage day-to-day activities. Moreover, the severity impairment of social communication and the presence of restricted and repetitive behaviors mirrored core ASD symptoms^55^. Children with ASD secondary to genetic syndromes, concomitant different neurological diseases, obesity, genetic and metabolic syndromes, immunodeficiencies, chronic diseases of the GI or respiratory tract, congenital cardiac defects, hepatic diseases, allergic diseases, food intolerances, use of antibiotics, pre-/pro- or synbiotics in the previous 4 weeks, were excluded. All subjects of these study did not present functional gastrointestinal disorders (i.e. constipation) according to Rome III criteria^56^.

The patients were enrolled at the Pediatric Unit of the University Hospital Federico II, Naples, Italy. The study was approved by the Ethics Committee of the University of Naples “Federico II”, Naples, Italy (N° 312/17). Informed consent was obtained from all legal guardians of the patients enrolled in the present study. All methods were performed in accordance with relevant guidelines and regulations.

### Host fecal DNA extraction

Host fecal DNA was extracted from frozen fecal samples using Qiagen QIAamp DNA Stool Mini Kit, following human DNA protocol. Briefly, ~ 150 mg of stools was homogenized, lysed and incubated at 70 °C for 10 min. The samples were then centrifuged to allow DNA to bind the column. After two washes, DNA was eluted in 100 μL of water. Extracted DNAs were checked for quality and quantity by spectrophotometric measurements with NanoDrop (ThermoFisher Scientific Inc) and stored at − 20 °C until processed.

### Host fecal DNA quantification

To quantify host fecal DNA in each sample, 1 μL of DNA was amplified trough Real Time-PCR using LightCycler 480 SYBR Green I Master (Roche Diagnostic) in a LightCycler480 RealTime thermocycler. The following protocol was adopted: 10 s for initial denaturation at 95 °C followed by 40 cycles consisting of 10 s at 94 °C for denaturation, 10 s at 60 °C for annealing, and 6 s for elongation at 72 °C temperature. The following primers were used for human GAPDH house-keeping gene: FW: 5’ CTGCAGTACTGTGGGGAGGT 3’; RV: 5’ CAAAGGCGGAGTTACCAGAG 3’.

### Methylome analyses

DNA methylation analyses were performed by using the Infinium MethylationEPIC array (Illumina, San Diego, California). As previously described^57^, 1000 ng of DNA per sample were bisulfite converted using the EZ DNA Methylation kit (Zymo Research), according to the manufacturer’s instructions. The bisulfite-treated DNA was hybridized onto the arrays and imaged with the iScan SQ instrument (Illumina). Array IDAT intensity data were preprocessed in R statistical environment using the RnBeads pipeline analysis package^58^. Methylation beta values ranging from 0 to 1 (corresponding to unmethylated to methylated signal intensity) for each sample were normalized using the methylumi package. Data were filtered by removing probes containing missing values, SNPs, and/or exhibiting low detection p-values (detection *p* value > 0.05). The obtained filtered beta values were then submitted to differential methylation analyses by using RnBeads package and differentially methylated CpG sites, promoters and genes were obtained. Differentially methylated sites and regions were classified as significant if the p-value was < 0.05.

### Gene Ontology Enrichment analysis of differentially methylated genes and promoters

Gene Ontology analysis for biological processes were conducted using the DAVID online software, using all human genes as background. Only genes and promoter significantly hypomethylated in ASD were included in the analysis, considering the few numbers of hypermethylated genes and promoters.

### Cell composition analysis

In order to identify the cell type origin of fecal DNA, two algorithms were applied: Leukocytes Unmethylation for Purity (LUMP)^59,60^ and MethylCIBERSORT, for the deconvolution of Treg cells, Neutrophils, Fibroblasts, Eosinophils, Endothelial cells, CD8, CD56, CD4, CD19 and CD14 cell types. LUMP algorithm was calculated within RnBeads package by screening 44 CpG sites particularly hypomethylated in leukocytes. MethylCIBERSORT package was downloaded and loaded into R environment to create reference matrices through limma-based feature selection^61^. These matrices were converted to text files and upload to CIBERSORTx online software to enumerate the proportions of distinct cell subpopulations^60^.

### Epigenetic age determination

The epigenetic age of ASD and CTRL children was determined by using RnBeads age prediction module that apply Horvath’s clock model^38^.

### Statistical analysis

All the statistical tests were performed using GraphPad Prism version 8.4.3 and R (version 4.1.3) (https://www.R-project.org/). Permutation test was computed to calculate the associations between the principal components and the disease considering significant *p* value < 0.01. Differentially methylated sites and regions were calculated by applying the limma^61^ linear model and adjusted using the empirical Bayes approach on derived M-values. Unpaired T test was used to compare HFD amount, Neutrophils Relative fraction and epigenetic age between CTRL and ASD groups. The criterion of statistical significance was set as a *p* value < 0.05.

### Supplementary Information

Supplementary Information 1.

Supplementary Figure S1.

Supplementary Table S1.

Supplementary Table S2.

## Data Availability

IDAT files with the relative annotation file have been submitted to BioStudies with the accession number S-BSST1180.
