# Supplementary material for: Host fecal DNA specific methylation signatures mark gut dysbiosis and inflammation in children affected by autism spectrum disorder
Source: Sci Rep. 2023 Oct 24;13:18197. doi: 10.1038/s41598-023-45132-0 (PMC10598023; doi:10.1038/s41598-023-45132-0)
Supplement: Supplementary file 1 — Supplementary Information 1. [file 41598_2023_45132_MOESM1_ESM.docx]

**Host fecal DNA specific methylation signatures mark gut dysbiosis and inflammation in children affected by autism spectrum disorder**

Mariella Cuomo^1,2^, Lorena Coretti^3^, Davide Costabile^2,4^, Rosa Della Monica^1,2^, Giulia De Riso^1^, Michela Buonaiuto^1,2^, Federica Trio^1,2^, Carmela Bravaccio^5^, Roberta Visconti^6^, Roberto Berni Canani^5^, Lorenzo Chiariotti^1,2,4*^ & Francesca Lembo^3*^

^1^Department of Molecular Medicine and Medical Biotechnologies, University of Naples “Federico II”, Via S. Pansini 5, 80131 Naples, Italy

^2^CEINGE Biotecnologie Avanzate “Franco Salvatore”, via G. Salvatore 482, 80145 Naples, Italy

^3^Department of Pharmacy, University of Naples “Federico II”, Via Domenico Montesano 49, 80131 Naples, Italy

^4^SEMM-European School of Molecular Medicine, University of Naples “Federico II”, Naples, Italy

^5^Department of Translational Medical Science - Pediatric Section, University of Naples Federico II, Naples, Italy.

^6^Institute for the Experimental Endocrinology and Oncology “G. Salvatore”, Italian National Council of Research, Via S. Pansini 5, 80131 Naples, Italy

Corresponding authors:

*Francesca Lembo: [frlembo@unina.it](mailto:frlembo@unina.it)

*Lorenzo Chiariotti: [chiariot@unina.it](mailto:chiariot@unina.it)

**Supplementary Information**

**Legend to Supplementary Figure S1.**

**Fecal microbial landscape in ASD children compared to non-affected children.** A) 3D PCoA obtained by Bray-Curtis distance matrix showing the separation of sample groups based on their gut microbial composition. B) Dot plot showing the top 20 features with significant differential abundance based on Linear Discriminant Analysis Effect Size (LEfSe; p > 0.05 for both Kruskal–Wallis and pairwise Wilcoxon tests and a cutoff value of LDA score above 2.0). Among the top 20 differently abundant OTUs, feature level analysis identified enrichment in gram-negative bacteria (Escherichia/Shigella coli and Bacteroides species) and an altered assortment in Firmicutes members in the microbiota of children with ASD. As belonging to the Firmicutes phylum, we found an increased level of the mucin-degrading bacterium Ruminococcus torques and high levels of Faecalibacterium prausnitzii in ASD patients at the expense of other colonizers of the gut microbiota in young children (Blautia and Streptococcus). C) Sankey plot displaying the number of predicted microbial pathways collapsed at the Super pathway level that are significantly enriched in each group as assessed by LEfSe algorithm (p > 0.05 for both Kruskal–Wallis and pairwise Wilcoxon tests and a cutoff value of LDA score above 2.0). Corresponding to the changes in several taxa, by applying Phylogenetic investigation of communities by reconstruction of unobserved states (PICRUSt) analysis to predict and estimate the functional content of the bacterial metagenome, here we found dissimilarities in the putative microbial metabolism of patients with ASD and CTRLs. Functional metabolic pathways, clustered in classes of “Infectious disease”, “Lipid metabolism”, “Glycan Biosynthesis and metabolism”, “Cellular process and signaling” and “Biosynthesis and biodegradation of secondary metabolites” were significantly enriched in ASD compared with CTRLs. Among these, the pathway of lipopolysaccharide biosynthesis, within the class of “Glycan Biosynthesis and metabolism”, was predominantly found in microbial communities of ASD group, possibly promoting an inflammatory signaling between microbes and the host. The PICRUSt prediction also indicated decreased metabolism of cofactors and vitamins (such as folate and thiamine metabolism) as well as metabolism of terpenoids and polyketides in microbial communities of ASD patients. These secondary metabolites by protecting cells and organs against damage, mutations, and reactive oxygen specie may have an important role in maintaining a healthy intestinal state. Interestingly, different assortment in functions involved in the metabolism of amino acids was observed in the two sample groups, suggesting that the microbial membership charactering gut microbiota of ASD children might influence intestinal amino acid availability and absorption. In particular, metabolism of alanine, aspartate, glutamate, cysteine, methionine, glycine serine and threonine and lysine biosynthesis were depleted in ASD while lysine degradation and phenylalanine metabolism were enriched. D) Heatmap showing Pearson’s correlation coefficients between the abundances of the top 20 differently abundant bacterial OTUs and the changes in the epigenetic clocks (blue: positive correlations; red: negative correlations; *p < 0.1, **p < 0.05).

**Supplementary Methods**

**Fecal microbiota sequencing and data analysis**. The V3-V4 16S rDNA FASTQ paired-end reads were obtained and pre-processed as previously described [8]. The rarefied operational taxonomic units (OTUs) table obtained with Quantitative Insights Into Microbial Ecology (QIIME, version 1.9.1) [S1] was imported in the MicrobiomeAnalyst package [S2] to remove rare OTUs, rarefy the samples to the minimum library size of 42,289 reads/sample, to evaluate beta diversity using Bray–Curtis at ASVs level followed by PERMANOVA analysis and to identify discriminatory OTUs by Linear Discriminant Analysis Effect Size (LEfSe) algorithm (p > 0.05 for both Kruskal–Wallis and pairwise Wilcoxon tests and a cutoff value of LDA score above 2.0) [S3].

We inferred the microbial gene content from the taxa abundance using PICRUSt [S4]. To estimate the accuracy of PICRUSt's prediction, the weighted Nearest Sequenced Taxon Index (NSTI) scores, which represent the phylogenetic distance for each OTU to its nearest sequenced reference bacterial genome, were calculated for each sample. The bacterial community samples had an average NSTI value < 0.06, thus PICRUSt provided useful functional predictions. Group differences in the inferred gene abundance of KEGG pathways (level-3) were identified by LEfSe (p < 0.05 by the Kruskal–Wallis test, p < 0.05 by the pairwise Wilcoxon test, and the logarithmic LDA score of 2.0) and summarized at Super Pathway (level-2) in a Sankey diagram created using SankeyMATIC (<https://sankeymatic.com>).

**Supplementary references**

[S1] Caporaso, J.G. et al. QIIME allows analysis of high-throughput community sequencing data. Nat. Methods. 7, 335-6 (2010).

[S2] Chong, J. et al. Using MicrobiomeAnalyst for comprehensive statistical, functional, and meta-analysis of microbiome data. Nat. Protoc. 15, 799–821 (2020).

[S3] Segata, N. et al. Metagenomic biomarker discovery and explanation. Genome Biol. 12, R60 (2011).

[S4] Langille, M. et al. Predictive functional profiling of microbial communities using 16S rRNA marker gene sequences. Nat. Biotechnol. 31, 814–821 (2013).
