# Supplementary figures and images for: Host fecal DNA specific methylation signatures mark gut dysbiosis and inflammation in children affected by autism spectrum disorder
Source: Sci Rep. 2023 Oct 24;13:18197. doi: 10.1038/s41598-023-45132-0 (PMC10598023; doi:10.1038/s41598-023-45132-0)

A

PERMANOVA p-value:0.02

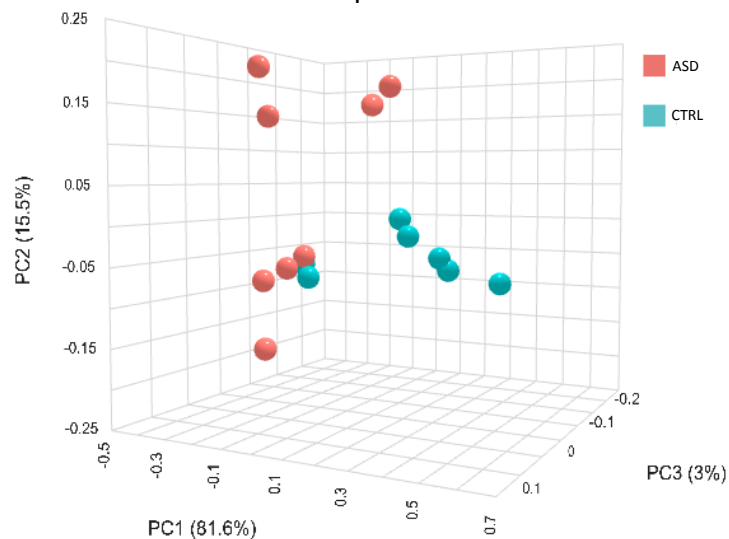

B

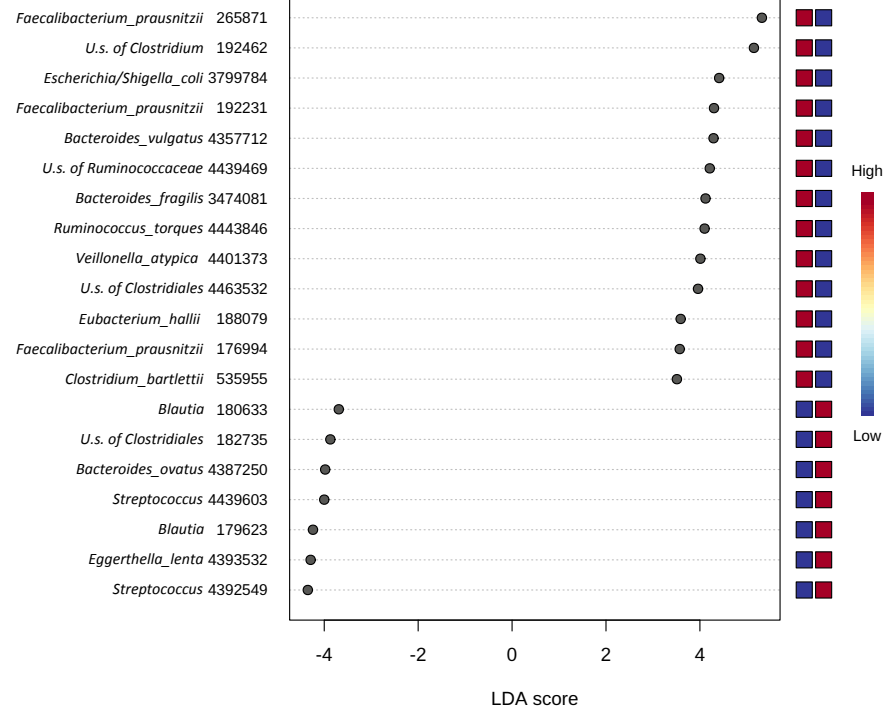

C

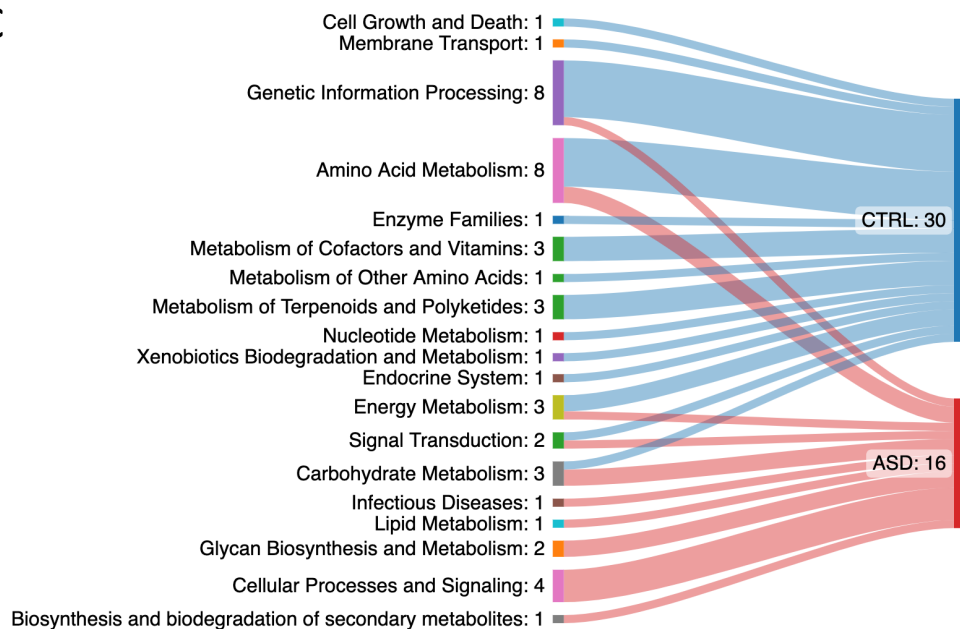

D

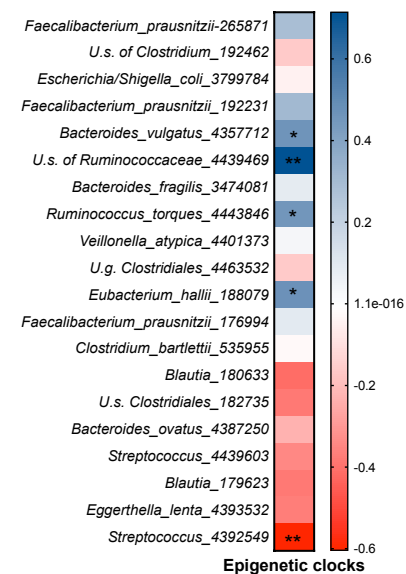

Supplement: Supplementary file 2 — Supplementary Figure S1. [file 41598_2023_45132_MOESM2_ESM.pdf]
