# Supplementary material for: Host fecal DNA specific methylation signatures mark gut dysbiosis and inflammation in children affected by autism spectrum disorder
Source: Sci Rep. 2023 Oct 24;13:18197. doi: 10.1038/s41598-023-45132-0 (PMC10598023; doi:10.1038/s41598-023-45132-0)
Supplement: Supplementary file 3 — Supplementary Table S1. [file 41598_2023_45132_MOESM3_ESM.docx]

**Supplementary Table 1** List of promoters hypermethylated in ASD patients.

| Gene Name | **Chromosome** | **Start** | **End** | **Number of sites** | **Average Methylation in CTRL** | **Average Methylation in ASD** | ***p*-value** |
| --- | --- | --- | --- | --- | --- | --- | --- |
| ACTN4P1 | 4 | 117517817 | 117519816 | 1 | 28,0% | 39,8% | 0,03 |
| ADPGK-AS1 | 15 | 73073676 | 73075675 | 1 | 25,1% | 34,5% | 0,04 |
| AHCYP1 | 10 | 47592879 | 47594878 | 1 | 24,8% | 38,9% | 0,02 |
| AHR | 7 | 17336746 | 17338745 | 2 | 16,0% | 28,6% | 0,02 |
| ALDH1A3 | 15 | 101416419 | 101418418 | 1 | 26,5% | 45,0% | 0,01 |
| ALDH1L1-AS1 | 3 | 125820983 | 125822982 | 2 | 32,2% | 35,3% | 0,02 |
| ANKRD20A18P | 21 | 15435146 | 15437145 | 1 | 14,4% | 21,9% | 0,03 |
| ANKRD20A19P | 13 | 24520422 | 24522421 | 1 | 24,7% | 38,1% | 0,05 |
| ANKRD36BP2 | 2 | 89063824 | 89065823 | 1 | 24,6% | 32,1% | 0,05 |
| ANP32BP1 | 15 | 75614535 | 75616534 | 1 | 24,7% | 32,0% | 0,05 |
| APBB2 | 4 | 41218232 | 41220231 | 1 | 16,4% | 23,8% | 0,04 |
| ARF1P3 | 8 | 125162664 | 125164663 | 1 | 20,8% | 28,8% | 0,05 |
| ARHGDIG | 16 | 317226 | 319225 | 3 | 34,7% | 39,4% | 0,01 |
| ARL5B | 10 | 18946834 | 18948833 | 5 | 24,5% | 32,5% | 0,05 |
| ARL5B-AS1 | 10 | 18947715 | 18949714 | 5 | 24,5% | 32,5% | 0,05 |
| ATP5L2 | 22 | 43036108 | 43038107 | 1 | 25,7% | 35,8% | 0,02 |
| ATP8B2 | 1 | 154296529 | 154298528 | 7 | 26,2% | 29,5% | 0,04 |
| BAIAP2L1 | 7 | 98029881 | 98031880 | 4 | 21,8% | 26,0% | 0,03 |
| BCKDHA | 19 | 41882715 | 41884714 | 1 | 22,9% | 35,7% | 0,01 |
| BET1 | 7 | 93633195 | 93635194 | 5 | 25,6% | 29,4% | 0,03 |
| BRINP1 | 9 | 122131246 | 122133245 | 3 | 26,6% | 32,3% | 0,05 |
| BTNL3 | 5 | 180414345 | 180416344 | 3 | 27,0% | 34,3% | 0,05 |
| C20orf62 | 20 | 43093485 | 43095484 | 1 | 30,6% | 44,2% | 0,003 |
| C3orf22 | 3 | 126277309 | 126279308 | 1 | 29,0% | 43,3% | 0,04 |
| C5AR1 | 19 | 47791780 | 47793779 | 1 | 24,9% | 33,9% | 0,05 |
| C9orf129 | 9 | 96108197 | 96110196 | 5 | 28,1% | 29,9% | 0,03 |
| C9orf147 | 9 | 115248985 | 115250984 | 3 | 26,0% | 29,2% | 0,04 |
| CA5A | 16 | 87969636 | 87971635 | 1 | 24,2% | 37,0% | 0,03 |
| CADM4 | 19 | 44143492 | 44145491 | 1 | 24,7% | 35,1% | 0,03 |
| CALCOCO2 | 17 | 46906850 | 46908849 | 3 | 25,2% | 28,2% | 0,02 |
| CARNS1 | 11 | 67180939 | 67182938 | 3 | 26,5% | 30,7% | 0,03 |
| CCDC182 | 17 | 55822174 | 55824173 | 2 | 23,0% | 30,1% | 0,03 |
| CCDC83 | 11 | 85564644 | 85566643 | 7 | 23,5% | 30,4% | 0,03 |
| CCL24 | 7 | 75452175 | 75454174 | 2 | 24,9% | 32,5% | 0,04 |
| CCRL2 | 3 | 46447154 | 46449153 | 5 | 25,4% | 30,9% | 0,05 |
| CD302 | 2 | 160654254 | 160656253 | 4 | 22,0% | 26,8% | 0,05 |
| CDC20B | 5 | 54468506 | 54470505 | 2 | 26,4% | 32,5% | 0,01 |
| CERCAM | 9 | 131172530 | 131174529 | 1 | 29,6% | 49,5% | 0,01 |
| CIB2 | 15 | 78423387 | 78425386 | 5 | 23,9% | 25,5% | 0,04 |
| CLDN6 | 16 | 3069573 | 3071572 | 4 | 24,3% | 26,9% | 0,05 |
| CMA1 | 14 | 24976972 | 24978971 | 1 | 25,4% | 33,0% | 0,05 |
| CNN2P8 | 2 | 95401582 | 95403581 | 1 | 16,4% | 29,4% | 0,01 |
| CNN3P1 | 6 | 6533430 | 6535429 | 1 | 20,7% | 32,2% | 0,03 |
| COL18A1 | 21 | 46823552 | 46825551 | 8 | 21,7% | 28,8% | 0,04 |
| CPB2 | 13 | 46678712 | 46680711 | 1 | 21,0% | 31,6% | 0,01 |
| CRYBA1 | 17 | 27572381 | 27574380 | 1 | 29,0% | 40,8% | 0,01 |
| CSF3 | 17 | 38170114 | 38172113 | 2 | 28,9% | 31,8% | 0,05 |
| CSRNP1 | 3 | 39195554 | 39197553 | 2 | 34,8% | 36,4% | 0,03 |
| CST9LP1 | 20 | 23530790 | 23532789 | 2 | 25,5% | 40,7% | 0,03 |
| CTBP2P6 | 11 | 43542086 | 43544085 | 1 | 26,6% | 44,9% | 0,01 |
| CYP17A1-AS1 | 10 | 104590978 | 104592977 | 2 | 26,6% | 31,6% | 0,04 |
| DCSTAMP | 8 | 105349815 | 105351814 | 2 | 32,5% | 36,4% | 0,03 |
| DDX11L2 | 2 | 114358645 | 114360644 | 3 | 28,3% | 35,6% | 0,03 |
| DENND3 | 8 | 142125877 | 142127876 | 1 | 19,4% | 29,8% | 0,01 |
| DIP2A-IT1 | 21 | 47880884 | 47882883 | 2 | 23,5% | 33,1% | 0,03 |
| DLEU7 | 13 | 51417576 | 51419575 | 5 | 26,6% | 29,2% | 0,05 |
| DMRT2 | 9 | 1048854 | 1050853 | 5 | 27,3% | 29,4% | 0,05 |
| DNAH10OS | 12 | 124419032 | 124421031 | 2 | 25,5% | 35,1% | 0,04 |
| DNAJC27-AS1 | 2 | 25192759 | 25194758 | 2 | 24,2% | 25,5% | 0,04 |
| DOPEY1 | 6 | 83775885 | 83777884 | 9 | 21,9% | 28,0% | 0,05 |
| DPY19L2P4 | 7 | 89747214 | 89749213 | 4 | 25,2% | 31,7% | 0,05 |
| DUSP11 | 2 | 74006785 | 74008784 | 4 | 26,7% | 29,6% | 0,04 |
| DYRK2 | 12 | 68040618 | 68042617 | 4 | 29,7% | 29,7% | 0,04 |
| ECHDC3 | 10 | 11782865 | 11784864 | 3 | 26,2% | 27,9% | 0,03 |
| EDF1 | 9 | 139760239 | 139762238 | 4 | 21,3% | 30,3% | 0,04 |
| EEF1DP2 | 9 | 95599451 | 95601450 | 1 | 32,3% | 43,2% | 0,02 |
| EIF2S2P3 | 10 | 94429001 | 94431000 | 1 | 21,3% | 34,2% | 0,03 |
| EMR4P | 19 | 6990358 | 6992357 | 1 | 23,2% | 31,6% | 0,05 |
| EOGT | 3 | 69062613 | 69064612 | 8 | 25,8% | 28,8% | 0,05 |
| ESPN | 1 | 6483348 | 6485347 | 6 | 29,0% | 31,6% | 0,04 |
| EVA1A | 2 | 75796349 | 75798348 | 1 | 29,2% | 36,7% | 0,04 |
| FAM3B | 21 | 42674639 | 42676638 | 2 | 20,8% | 28,4% | 0,05 |
| FAM83C | 20 | 33879705 | 33881704 | 4 | 32,4% | 37,6% | 0,05 |
| FREM1 | 9 | 14910494 | 14912493 | 1 | 31,5% | 42,4% | 0,03 |
| GINM1 | 6 | 149885930 | 149887929 | 2 | 21,1% | 24,9% | 0,03 |
| GOLPH3 | 5 | 32173957 | 32175956 | 6 | 22,4% | 27,9% | 0,05 |
| GPR78 | 4 | 8558952 | 8560951 | 2 | 25,8% | 36,3% | 0,04 |
| GRAMD4 | 22 | 46970409 | 46972408 | 2 | 27,0% | 36,5% | 0,04 |
| GUCA1A | 6 | 42121644 | 42123643 | 2 | 29,8% | 30,5% | 0,04 |
| H2AFY2 | 10 | 71811052 | 71813051 | 2 | 22,7% | 25,6% | 0,01 |
| HBP1 | 7 | 106807906 | 106809905 | 2 | 17,1% | 26,6% | 0,03 |
| HES2 | 1 | 6484231 | 6486230 | 5 | 29,3% | 32,2% | 0,02 |
| HIBCH | 2 | 191208420 | 191210419 | 3 | 18,1% | 25,3% | 0,05 |
| HIGD1AP9 | 12 | 50011617 | 50013616 | 2 | 25,7% | 32,8% | 0,03 |
| HMGB1P8 | 15 | 89679227 | 89681226 | 1 | 22,1% | 33,9% | 0,01 |
| HPS6 | 10 | 103823647 | 103825646 | 3 | 25,0% | 32,2% | 0,04 |
| HS3ST4 | 16 | 25701847 | 25703846 | 4 | 31,7% | 33,3% | 0,01 |
| HSD17B11 | 4 | 88312039 | 88314038 | 5 | 24,2% | 29,0% | 0,05 |
| HSPA7 | 1 | 161574581 | 161576580 | 1 | 27,9% | 40,6% | 0,02 |
| HTATSF1P2 | 6 | 3023507 | 3025506 | 4 | 28,7% | 30,5% | 0,04 |
| IDH3A | 15 | 78422340 | 78424339 | 4 | 21,9% | 25,7% | 0,03 |
| IGHD | 14 | 106311511 | 106313510 | 1 | 30,4% | 46,2% | 0,02 |
| IGLC5 | 22 | 23256305 | 23258304 | 1 | 23,9% | 33,2% | 0,01 |
| IGLC6 | 22 | 23260207 | 23262206 | 1 | 18,5% | 33,7% | 0,003 |
| IGLJ5 | 22 | 23254908 | 23256907 | 1 | 23,9% | 33,2% | 0,01 |
| IGLJ6 | 22 | 23258804 | 23260803 | 2 | 26,9% | 35,2% | 0,05 |
| IGLV2-5 | 22 | 23197437 | 23199436 | 1 | 29,6% | 42,1% | 0,03 |
| IGLV3-25 | 22 | 23027690 | 23029689 | 1 | 25,5% | 35,8% | 0,04 |
| IL18BP | 11 | 71708087 | 71710086 | 1 | 27,1% | 36,6% | 0,05 |
| INHA | 2 | 220432384 | 220434383 | 1 | 26,1% | 38,0% | 0,01 |
| INPP5K | 17 | 1419683 | 1421682 | 9 | 26,9% | 28,1% | 0,05 |
| IRGC | 19 | 44218662 | 44220661 | 4 | 30,7% | 35,1% | 0,04 |
| IRGM | 5 | 150224585 | 150226584 | 1 | 33,3% | 47,9% | 0,02 |
| KIAA0391 | 14 | 35589552 | 35591551 | 3 | 23,0% | 30,9% | 0,03 |
| KRR1 | 12 | 75904917 | 75906916 | 3 | 31,9% | 33,0% | 0,04 |
| KRT121P | 12 | 52652152 | 52654151 | 1 | 23,5% | 34,9% | 0,04 |
| KRT38 | 17 | 39596674 | 39598673 | 1 | 19,8% | 28,8% | 0,04 |
| KRT77 | 12 | 53096748 | 53098747 | 1 | 24,6% | 32,5% | 0,03 |
| KRT8P26 | 11 | 65492910 | 65494909 | 1 | 30,8% | 40,8% | 0,04 |
| KRTAP5-9 | 11 | 71257966 | 71259965 | 3 | 30,6% | 35,4% | 0,05 |
| LAMB3 | 1 | 209825312 | 209827311 | 1 | 29,2% | 40,4% | 0,04 |
| LINC00350 | 13 | 20159758 | 20161757 | 3 | 18,5% | 27,6% | 0,01 |
| LINC00355 | 13 | 64649645 | 64651644 | 2 | 24,7% | 33,1% | 0,02 |
| LINC00400 | 13 | 43731267 | 43733266 | 1 | 22,9% | 31,8% | 0,02 |
| LINC00614 | 10 | 27232848 | 27234847 | 1 | 15,4% | 34,2% | 0,0004 |
| LINC00639 | 14 | 39416978 | 39418977 | 1 | 26,2% | 36,9% | 0,03 |
| LINC00669 | 18 | 37379783 | 37381782 | 1 | 30,8% | 41,5% | 0,05 |
| LINC00908 | 18 | 74239112 | 74241111 | 2 | 30,9% | 35,4% | 0,02 |
| MAK | 6 | 10838265 | 10840264 | 6 | 25,9% | 27,3% | 0,05 |
| MBD5 | 2 | 148777080 | 148779079 | 1 | 29,6% | 38,9% | 0,04 |
| MIR151A | 8 | 141742253 | 141744252 | 1 | 21,2% | 31,3% | 0,01 |
| MIR1827 | 15 | 78329373 | 78331372 | 1 | 29,6% | 48,2% | 0,0004 |
| MIR3185 | 17 | 46801338 | 46803337 | 4 | 28,3% | 30,5% | 0,04 |
| MIR324 | 17 | 7126199 | 7128198 | 1 | 19,9% | 32,2% | 0,0003 |
| MIR335 | 7 | 130134452 | 130136451 | 1 | 23,8% | 30,9% | 0,03 |
| MIR3646 | 20 | 43035260 | 43037259 | 1 | 20,4% | 30,9% | 0,005 |
| MIR4468 | 7 | 137807004 | 137809003 | 1 | 27,9% | 39,1% | 0,01 |
| MIR449C | 5 | 54467682 | 54469681 | 2 | 26,4% | 32,5% | 0,01 |
| MIR4525 | 17 | 80625684 | 80627683 | 1 | 28,3% | 44,6% | 0,002 |
| MIR4535 | 22 | 49174607 | 49176606 | 1 | 23,1% | 39,0% | 0,004 |
| MIR4632 | 1 | 12250270 | 12252269 | 2 | 27,9% | 39,4% | 0,03 |
| MIR4656 | 7 | 4827771 | 4829770 | 5 | 29,2% | 40,3% | 0,04 |
| MIR4749 | 19 | 50356348 | 50358347 | 1 | 25,1% | 37,4% | 0,01 |
| MIR4758 | 20 | 60907114 | 60909113 | 2 | 32,3% | 42,7% | 0,04 |
| MIR4777 | 2 | 232225919 | 232227918 | 1 | 25,7% | 32,7% | 0,05 |
| MIR4800 | 4 | 2251384 | 2253383 | 3 | 31,2% | 33,7% | 0,03 |
| MIR585 | 5 | 168690199 | 168692198 | 1 | 24,9% | 32,9% | 0,04 |
| MIR629 | 15 | 70371308 | 70373307 | 1 | 27,0% | 34,7% | 0,02 |
| MIRLET7D | 9 | 96939616 | 96941615 | 1 | 27,1% | 37,8% | 0,03 |
| MLLT1 | 19 | 6279460 | 6281459 | 2 | 23,7% | 28,2% | 0,05 |
| MOB2 | 11 | 1521978 | 1523977 | 2 | 31,3% | 40,6% | 0,04 |
| MPV17 | 2 | 27548048 | 27550047 | 1 | 31,1% | 44,7% | 0,04 |
| MRGPRG | 11 | 3239544 | 3241543 | 7 | 27,6% | 33,7% | 0,02 |
| MRPL23-AS1 | 11 | 2010651 | 2012650 | 8 | 25,6% | 33,6% | 0,03 |
| MT4 | 16 | 56597461 | 56599460 | 2 | 23,5% | 30,7% | 0,02 |
| NDUFAF4P2 | 7 | 93472601 | 93474600 | 1 | 21,8% | 34,2% | 0,001 |
| NEU4 | 2 | 242748420 | 242750419 | 4 | 27,5% | 38,7% | 0,02 |
| NFATC4 | 14 | 24833379 | 24835378 | 2 | 22,3% | 26,9% | 0,04 |
| NFIX | 19 | 13104922 | 13106921 | 3 | 31,5% | 33,2% | 0,04 |
| NHP2L1 | 22 | 42086009 | 42088008 | 2 | 25,3% | 33,2% | 0,05 |
| NIFK | 2 | 122494000 | 122495999 | 5 | 28,9% | 32,1% | 0,04 |
| NINJ2 | 12 | 772446 | 774445 | 1 | 29,1% | 41,8% | 0,05 |
| NMUR2 | 5 | 151812430 | 151814429 | 1 | 11,4% | 24,8% | 0,003 |
| NOS2P1 | 17 | 25992239 | 25994238 | 1 | 20,8% | 31,4% | 0,03 |
| NPC1L1 | 7 | 44580415 | 44582414 | 9 | 30,0% | 38,8% | 0,05 |
| NT5DC4 | 2 | 113477563 | 113479562 | 3 | 26,4% | 40,3% | 0,003 |
| NUDT16P1 | 3 | 131079242 | 131081241 | 5 | 32,7% | 32,9% | 0,05 |
| NXNL2 | 9 | 91148516 | 91150515 | 4 | 23,9% | 30,4% | 0,05 |
| OCIAD1-AS1 | 4 | 48861721 | 48863720 | 1 | 15,2% | 24,6% | 0,01 |
| ODF2-AS1 | 9 | 131234792 | 131236791 | 1 | 26,7% | 37,9% | 0,01 |
| OPLAH | 8 | 145118236 | 145120235 | 2 | 27,8% | 28,1% | 0,05 |
| OR10D5P | 11 | 123924077 | 123926076 | 1 | 26,3% | 34,8% | 0,03 |
| ORAI2 | 7 | 102072053 | 102074052 | 4 | 25,2% | 30,1% | 0,04 |
| OTOG | 11 | 17567420 | 17569419 | 4 | 26,0% | 29,1% | 0,05 |
| PAM16 | 16 | 4405109 | 4407108 | 3 | 31,4% | 37,3% | 0,02 |
| PARP1P1 | 13 | 111587606 | 111589605 | 2 | 23,9% | 32,1% | 0,04 |
| PCDH9-AS1 | 13 | 66876503 | 66878502 | 1 | 18,0% | 27,3% | 0,05 |
| PI4KAP2 | 22 | 21871323 | 21873322 | 1 | 23,9% | 33,0% | 0,03 |
| PIGPP3 | 20 | 32551468 | 32553467 | 1 | 15,2% | 27,9% | 0,001 |
| PIM1 | 6 | 37136479 | 37138478 | 4 | 18,2% | 26,8% | 0,04 |
| PIP | 7 | 142827670 | 142829669 | 2 | 28,2% | 31,7% | 0,05 |
| PLA2G6 | 22 | 38601198 | 38603197 | 1 | 24,8% | 31,8% | 0,05 |
| POLD2 | 7 | 44163458 | 44165457 | 1 | 18,6% | 33,6% | 0,003 |
| POLD2P1 | 5 | 92601635 | 92603634 | 2 | 26,9% | 36,9% | 0,02 |
| PPP1R11P1 | 1 | 21722980 | 21724979 | 1 | 20,3% | 32,1% | 0,03 |
| PPP1R14BP5 | 6 | 126579002 | 126581001 | 1 | 25,0% | 34,5% | 0,05 |
| PPP2CB | 8 | 30671331 | 30673330 | 1 | 26,3% | 34,5% | 0,02 |
| PRLH | 2 | 238473717 | 238475716 | 3 | 35,0% | 38,1% | 0,01 |
| PRM2 | 16 | 11369838 | 11371837 | 4 | 28,8% | 38,2% | 0,04 |
| PRND | 20 | 4701056 | 4703055 | 3 | 30,2% | 31,2% | 0,05 |
| PROB1 | 5 | 138730386 | 138732385 | 2 | 30,0% | 42,3% | 0,05 |
| PRSS55 | 8 | 10381556 | 10383555 | 3 | 31,6% | 36,7% | 0,04 |
| PRUNE2 | 9 | 79520504 | 79522503 | 11 | 28,3% | 28,9% | 0,03 |
| PSME4 | 2 | 54197478 | 54199477 | 3 | 25,1% | 28,1% | 0,04 |
| PSPN | 19 | 6378570 | 6380569 | 1 | 28,1% | 36,1% | 0,05 |
| PTER | 10 | 16477442 | 16479441 | 2 | 24,3% | 27,6% | 0,02 |
| PTGES3P3 | 4 | 170712354 | 170714353 | 1 | 16,3% | 27,0% | 0,01 |
| PTGS1 | 9 | 125131324 | 125133323 | 3 | 26,7% | 27,8% | 0,03 |
| RAB3IL1 | 11 | 61687242 | 61689241 | 4 | 29,2% | 37,9% | 0,03 |
| RAB5B | 12 | 56366197 | 56368196 | 6 | 26,5% | 29,1% | 0,05 |
| RCC2P3 | 7 | 137805448 | 137807447 | 1 | 27,9% | 39,1% | 0,01 |
| RMDN2 | 2 | 38148830 | 38150829 | 1 | 25,2% | 33,7% | 0,02 |
| RMRPP1 | 10 | 75533800 | 75535799 | 1 | 23,3% | 33,0% | 0,05 |
| RN7SL22P | 9 | 35670273 | 35672272 | 2 | 32,7% | 33,7% | 0,01 |
| RN7SL704P | 22 | 38626226 | 38628225 | 3 | 33,8% | 34,4% | 0,05 |
| RN7SL750P | 7 | 100418387 | 100420386 | 1 | 24,9% | 36,2% | 0,05 |
| RN7SL775P | 17 | 17080229 | 17082228 | 1 | 28,9% | 41,5% | 0,04 |
| RN7SL871P | 17 | 39993081 | 39995080 | 1 | 23,5% | 35,7% | 0,01 |
| RNA5-8SP2 | 16 | 33963926 | 33965925 | 4 | 25,3% | 32,6% | 0,04 |
| RNA5SP23 | 1 | 78840454 | 78842453 | 1 | 22,9% | 32,6% | 0,02 |
| RNA5SP440 | 17 | 36887674 | 36889673 | 2 | 26,8% | 39,2% | 0,01 |
| RNA5SP464 | 19 | 12106124 | 12108123 | 1 | 18,0% | 26,9% | 0,01 |
| RNA5SP88 | 2 | 24787108 | 24789107 | 1 | 29,7% | 42,4% | 0,04 |
| RNMTL1P1 | 10 | 21681462 | 21683461 | 1 | 21,2% | 32,5% | 0,03 |
| RNU1-91P | 11 | 3069252 | 3071251 | 1 | 26,2% | 33,3% | 0,04 |
| RNU2-25P | 9 | 2162454 | 2164453 | 1 | 18,8% | 32,6% | 0,03 |
| RNU6-1251P | 20 | 42100153 | 42102152 | 1 | 19,7% | 28,8% | 0,003 |
| RNU6-178P | 8 | 28272465 | 28274464 | 1 | 15,3% | 24,9% | 0,03 |
| RNU6-179P | 1 | 154011999 | 154013998 | 1 | 23,6% | 35,2% | 0,05 |
| RNU6-195P | 19 | 41213569 | 41215568 | 1 | 23,7% | 32,7% | 0,05 |
| RNU6-371P | 1 | 62763429 | 62765428 | 1 | 19,4% | 33,7% | 0,003 |
| RNU6-40P | 1 | 31970026 | 31972025 | 1 | 18,2% | 25,5% | 0,02 |
| RNU6-482P | 5 | 112112560 | 112114559 | 1 | 27,9% | 37,6% | 0,05 |
| RNU6-499P | 4 | 83094200 | 83096199 | 1 | 26,5% | 37,4% | 0,01 |
| RNU6-601P | 2 | 150466789 | 150468788 | 2 | 23,8% | 34,0% | 0,04 |
| RNU6-718P | 5 | 119672849 | 119674848 | 1 | 22,2% | 32,5% | 0,01 |
| RNU6-75P | 13 | 91433624 | 91435623 | 1 | 21,9% | 34,6% | 0,02 |
| RNU6-774P | 4 | 85153310 | 85155309 | 1 | 22,5% | 30,3% | 0,04 |
| RNU6-973P | 7 | 65324254 | 65326253 | 1 | 27,2% | 37,6% | 0,04 |
| RNU7-127P | 2 | 236944394 | 236946393 | 1 | 24,8% | 38,3% | 0,01 |
| RNU7-136P | 3 | 160189705 | 160191704 | 1 | 23,0% | 30,4% | 0,05 |
| RNU7-67P | 8 | 102318095 | 102320094 | 1 | 29,6% | 46,0% | 0,02 |
| RNY3P9 | 13 | 40799464 | 40801463 | 1 | 17,2% | 28,0% | 0,05 |
| RPL35P9 | 13 | 107076621 | 107078620 | 1 | 31,7% | 45,1% | 0,01 |
| RPS11P5 | 12 | 133400787 | 133402786 | 2 | 29,5% | 35,5% | 0,04 |
| RPS2P32 | 7 | 23528592 | 23530591 | 1 | 17,4% | 24,7% | 0,05 |
| RRAS2 | 11 | 14385553 | 14387552 | 1 | 20,4% | 28,7% | 0,05 |
| RTN4R | 22 | 20270270 | 20272269 | 1 | 29,0% | 41,3% | 0,03 |
| RWDD4P1 | 7 | 104940469 | 104942468 | 1 | 28,1% | 35,4% | 0,04 |
| SBNO1 | 12 | 123848891 | 123850890 | 1 | 12,1% | 26,4% | 0,03 |
| SCARB1 | 12 | 125366715 | 125368714 | 2 | 27,4% | 36,2% | 0,04 |
| SCARNA12 | 12 | 7076270 | 7078269 | 3 | 26,0% | 36,3% | 0,05 |
| SEC31A | 4 | 83821820 | 83823819 | 2 | 24,2% | 24,7% | 0,05 |
| SERPINA10 | 14 | 94759109 | 94761108 | 3 | 27,8% | 33,2% | 0,05 |
| SERPINB11 | 18 | 61313313 | 61315312 | 1 | 25,8% | 35,8% | 0,04 |
| SETX | 9 | 135229873 | 135231872 | 2 | 22,4% | 35,0% | 0,03 |
| SIAH2 | 3 | 150480765 | 150482764 | 6 | 27,4% | 27,8% | 0,05 |
| SLA | 8 | 134114799 | 134116798 | 2 | 28,6% | 29,5% | 0,05 |
| SLC18A2 | 10 | 118999104 | 119001103 | 1 | 24,5% | 38,2% | 0,002 |
| SLC26A1 | 4 | 986729 | 988728 | 5 | 28,0% | 36,3% | 0,04 |
| SLC34A3 | 9 | 140123709 | 140125708 | 7 | 28,7% | 38,4% | 0,05 |
| SMCR2 | 17 | 17580503 | 17582502 | 1 | 28,9% | 36,4% | 0,05 |
| SNORA25 | 11 | 93463313 | 93465312 | 1 | 24,0% | 35,7% | 0,05 |
| SNORD114-25 | 14 | 101450894 | 101452893 | 1 | 18,9% | 29,3% | 0,004 |
| SNORD115-15 | 15 | 25441223 | 25443222 | 2 | 30,8% | 39,5% | 0,05 |
| SNORD115-6 | 15 | 25424144 | 25426143 | 2 | 27,4% | 39,1% | 0,02 |
| SNORD116-8 | 15 | 25314079 | 25316078 | 5 | 39,6% | 44,3% | 0,04 |
| SNORD88A | 19 | 51302290 | 51304289 | 1 | 24,6% | 38,4% | 0,01 |
| SNRPGP5 | 10 | 13146294 | 13148293 | 2 | 15,3% | 26,9% | 0,01 |
| SPATA3 | 2 | 231859336 | 231861335 | 4 | 31,6% | 31,9% | 0,04 |
| SPATA31E1 | 9 | 90496241 | 90498240 | 1 | 26,7% | 44,3% | 0,001 |
| SPCS2P3 | 5 | 814961 | 816960 | 1 | 16,7% | 30,5% | 0,004 |
| SYBU | 8 | 110703521 | 110705520 | 6 | 29,6% | 32,1% | 0,05 |
| TAAR6 | 6 | 132889961 | 132891960 | 2 | 26,5% | 29,1% | 0,04 |
| TAMM41 | 3 | 11887894 | 11889893 | 2 | 22,0% | 25,1% | 0,05 |
| TAS1R3 | 1 | 1265194 | 1267193 | 4 | 29,5% | 38,5% | 0,05 |
| TBCEL | 11 | 120893281 | 120895280 | 4 | 29,0% | 29,5% | 0,03 |
| TCEB2P2 | 11 | 129902616 | 129904615 | 3 | 25,4% | 31,1% | 0,02 |
| TDGF1P2 | 2 | 228734861 | 228736860 | 2 | 31,3% | 32,8% | 0,04 |
| TMEM126A | 11 | 85357511 | 85359510 | 3 | 24,0% | 28,9% | 0,05 |
| TMEM86B | 19 | 55741148 | 55743147 | 2 | 28,2% | 35,9% | 0,05 |
| TMEM8C | 9 | 136393235 | 136395234 | 3 | 25,0% | 35,1% | 0,03 |
| TMTC1 | 12 | 29937193 | 29939192 | 1 | 20,5% | 30,6% | 0,01 |
| TNNI1 | 1 | 201398495 | 201400494 | 1 | 28,1% | 36,6% | 0,02 |
| TOMM22P5 | 10 | 120401772 | 120403771 | 1 | 23,9% | 34,0% | 0,02 |
| TP53I13 | 17 | 27891570 | 27893569 | 2 | 23,9% | 29,3% | 0,02 |
| TRAV10 | 14 | 22292136 | 22294135 | 1 | 27,6% | 37,7% | 0,05 |
| TSACC | 1 | 156305605 | 156307604 | 1 | 19,4% | 29,7% | 0,03 |
| TSHZ2 | 20 | 51587446 | 51589445 | 1 | 18,4% | 30,0% | 0,01 |
| TUBGCP2 | 10 | 135125342 | 135127341 | 2 | 24,4% | 32,8% | 0,04 |
| TVP23C | 17 | 15466410 | 15468409 | 1 | 15,0% | 25,1% | 0,02 |
| TVP23C-CDRT4 | 17 | 15466376 | 15468375 | 1 | 15,0% | 25,1% | 0,02 |
| UBA52P5 | 8 | 124247592 | 124249591 | 1 | 22,8% | 34,2% | 0,01 |
| ULBP1 | 6 | 150283643 | 150285642 | 3 | 28,9% | 30,1% | 0,04 |
| USP33 | 1 | 78225038 | 78227037 | 2 | 27,5% | 35,5% | 0,04 |
| VIPR2 | 7 | 158937150 | 158939149 | 2 | 24,7% | 35,1% | 0,04 |
| WASF3-AS1 | 13 | 27215002 | 27217001 | 1 | 16,2% | 26,6% | 0,04 |
| ZBTB46 | 20 | 62462098 | 62464097 | 1 | 22,0% | 34,8% | 0,004 |
| ZCCHC6 | 9 | 88968870 | 88970869 | 6 | 23,5% | 29,6% | 0,02 |
| ZDHHC20P3 | 11 | 74939203 | 74941202 | 1 | 26,7% | 35,2% | 0,04 |
| ZFP64P1 | 14 | 51171730 | 51173729 | 2 | 31,9% | 33,3% | 0,05 |
| ZNF143 | 11 | 9480366 | 9482365 | 5 | 26,8% | 28,1% | 0,04 |
| ZNF404 | 19 | 44405038 | 44407037 | 2 | 29,0% | 30,8% | 0,05 |
| ZNF677 | 19 | 53757652 | 53759651 | 4 | 20,8% | 29,4% | 0,03 |
| ZNF732 | 4 | 298611 | 300610 | 1 | 19,9% | 34,7% | 0,01 |
| ZNF783 | 7 | 148957762 | 148959761 | 2 | 20,5% | 27,6% | 0,01 |
| ZNF8 | 19 | 58788818 | 58790817 | 1 | 19,9% | 35,8% | 0,02 |
| ZNF852 | 3 | 44551629 | 44553628 | 3 | 26,9% | 29,4% | 0,04 |
