# Supplementary material for: Host fecal DNA specific methylation signatures mark gut dysbiosis and inflammation in children affected by autism spectrum disorder
Source: Sci Rep. 2023 Oct 24;13:18197. doi: 10.1038/s41598-023-45132-0 (PMC10598023; doi:10.1038/s41598-023-45132-0)
Supplement: Supplementary file 4 — Supplementary Table S2. [file 41598_2023_45132_MOESM4_ESM.docx]

**Supplementary Table 2** List of promoters hypomethylated in ASD patients.

| Gene Name | **Chromosome** | **Start** | **End** | **Number of sites** | **Average Methylation in CTRL** | **Average Methylation in ASD** | ***p*-value** |
| --- | --- | --- | --- | --- | --- | --- | --- |
| A2M | 12 | 9268326 | 9270325 | 1 | 50,7% | 32,1% | 0,001 |
| ABCA10 | 17 | 67240488 | 67242487 | 1 | 38,9% | 27,7% | 0,02 |
| ABCC11 | 16 | 48280980 | 48282979 | 1 | 51,8% | 36,6% | 0,02 |
| ABCD1P5 | 2 | 92027127 | 92029126 | 1 | 29,6% | 22,1% | 0,05 |
| ABLIM2 | 4 | 8160060 | 8162059 | 7 | 29,6% | 24,4% | 0,03 |
| ACAP2-IT1 | 3 | 195002971 | 195004970 | 1 | 44,2% | 34,1% | 0,04 |
| ACBD6 | 1 | 180471590 | 180473589 | 3 | 30,3% | 27,2% | 0,05 |
| ACKR3 | 2 | 237474930 | 237476929 | 1 | 33,8% | 22,6% | 0,03 |
| ADAR | 1 | 154599976 | 154601975 | 2 | 30,3% | 23,3% | 0,05 |
| AFAP1 | 4 | 7941154 | 7943153 | 2 | 47,4% | 37,3% | 0,04 |
| AGBL1 | 15 | 86683727 | 86685726 | 2 | 38,9% | 33,8% | 0,05 |
| ALDH3A2 | 17 | 19549949 | 19551948 | 5 | 27,0% | 26,0% | 0,05 |
| ALKBH3 | 11 | 43900861 | 43902860 | 4 | 33,1% | 25,1% | 0,05 |
| ANGPTL4 | 19 | 8426673 | 8428672 | 2 | 45,2% | 33,2% | 0,01 |
| ANK1 | 8 | 41753781 | 41755780 | 3 | 27,3% | 26,5% | 0,03 |
| ANKRD27 | 19 | 33167004 | 33169003 | 3 | 28,3% | 27,2% | 0,04 |
| AP5S1 | 20 | 3799678 | 3801677 | 9 | 34,7% | 25,4% | 0,04 |
| AQP8 | 16 | 25225552 | 25227551 | 1 | 32,3% | 23,7% | 0,02 |
| ARHGAP20 | 11 | 110583413 | 110585412 | 4 | 42,3% | 31,0% | 0,03 |
| ARHGEF38-IT1 | 4 | 106481248 | 106483247 | 1 | 43,5% | 31,7% | 0,04 |
| ARL13B | 3 | 93697483 | 93699482 | 5 | 31,7% | 24,7% | 0,03 |
| ARMC1 | 8 | 66545943 | 66547942 | 2 | 28,8% | 27,2% | 0,03 |
| ATF6 | 1 | 161734584 | 161736583 | 4 | 38,4% | 29,8% | 0,05 |
| ATG12P2 | 2 | 223769274 | 223771273 | 1 | 56,3% | 37,4% | 0,001 |
| ATHL1 | 11 | 287635 | 289634 | 3 | 30,7% | 29,4% | 0,01 |
| B3GALT6 | 1 | 1166129 | 1168128 | 2 | 28,8% | 24,7% | 0,02 |
| BCAR4 | 16 | 11922203 | 11924202 | 1 | 46,1% | 28,7% | 0,003 |
| BNIP3L | 8 | 26238914 | 26240913 | 7 | 31,9% | 27,2% | 0,02 |
| C10orf111 | 10 | 15138819 | 15140818 | 6 | 32,6% | 24,0% | 0,05 |
| C10orf12 | 10 | 98739541 | 98741540 | 1 | 35,8% | 24,1% | 0,01 |
| C16orf71 | 16 | 4782773 | 4784772 | 8 | 29,4% | 26,9% | 0,03 |
| C16orf74 | 16 | 85784236 | 85786235 | 4 | 31,3% | 27,8% | 0,05 |
| C16orf92 | 16 | 30033155 | 30035154 | 2 | 29,4% | 28,5% | 0,03 |
| C19orf84 | 19 | 51893329 | 51895328 | 2 | 35,5% | 29,9% | 0,03 |
| C1orf180 | 1 | 85100204 | 85102203 | 1 | 50,8% | 36,4% | 0,02 |
| C21orf91 | 21 | 19191204 | 19193203 | 4 | 29,9% | 25,1% | 0,01 |
| C2orf72 | 2 | 231900705 | 231902704 | 2 | 33,8% | 25,1% | 0,04 |
| C5orf17 | 5 | 23949957 | 23951956 | 1 | 39,0% | 27,4% | 0,03 |
| C8orf34 | 8 | 69241457 | 69243456 | 1 | 51,6% | 37,1% | 0,01 |
| CALU | 7 | 128377846 | 128379845 | 7 | 31,6% | 27,6% | 0,03 |
| CASP8AP2 | 6 | 90538113 | 90540112 | 2 | 32,2% | 25,0% | 0,04 |
| CATSPERD | 19 | 5719188 | 5721187 | 10 | 33,9% | 28,3% | 0,05 |
| CAV2 | 7 | 115925934 | 115927933 | 1 | 49,7% | 31,8% | 0,0001 |
| CBX7 | 22 | 39548180 | 39550179 | 5 | 40,4% | 30,6% | 0,02 |
| CCNC | 6 | 100016350 | 100018349 | 4 | 44,6% | 35,6% | 0,05 |
| CCR3 | 3 | 46203596 | 46205595 | 1 | 41,2% | 31,0% | 0,04 |
| CD300LF | 17 | 72708618 | 72710617 | 3 | 40,6% | 30,9% | 0,03 |
| CDK12 | 17 | 37616264 | 37618263 | 8 | 37,9% | 29,8% | 0,05 |
| CEACAM3 | 19 | 42298869 | 42300868 | 3 | 37,1% | 30,4% | 0,05 |
| CFTR | 7 | 117104338 | 117106337 | 1 | 41,3% | 29,5% | 0,004 |
| CHAD | 17 | 48545828 | 48547827 | 8 | 35,3% | 28,2% | 0,05 |
| CHSY1 | 15 | 101791638 | 101793637 | 3 | 36,0% | 24,4% | 0,01 |
| CKAP4 | 12 | 106697558 | 106699557 | 1 | 33,9% | 23,6% | 0,03 |
| CLDN10 | 13 | 96084358 | 96086357 | 4 | 34,6% | 29,5% | 0,05 |
| CLDN10-AS1 | 13 | 96185665 | 96187664 | 1 | 33,6% | 25,1% | 0,05 |
| CLIP3 | 19 | 36523746 | 36525745 | 3 | 29,1% | 28,0% | 0,05 |
| CLTA | 9 | 36189353 | 36191352 | 4 | 30,8% | 23,4% | 0,04 |
| COL23A1 | 5 | 178017057 | 178019056 | 5 | 33,4% | 26,8% | 0,05 |
| COL3A1 | 2 | 189837546 | 189839545 | 2 | 40,3% | 28,7% | 0,03 |
| COX6CP6 | 3 | 70798774 | 70800773 | 1 | 36,5% | 26,1% | 0,03 |
| CPA5 | 7 | 129983130 | 129985129 | 1 | 34,9% | 24,0% | 0,03 |
| CRISP1 | 6 | 49844310 | 49846309 | 1 | 34,8% | 22,6% | 0,01 |
| CRISPLD2 | 16 | 84852090 | 84854089 | 3 | 32,7% | 32,4% | 0,05 |
| CWF19L1 | 10 | 102026938 | 102028937 | 3 | 34,1% | 31,1% | 0,05 |
| CXCL13 | 4 | 78431407 | 78433406 | 1 | 28,0% | 19,8% | 0,03 |
| CXCR4 | 2 | 136875236 | 136877235 | 4 | 27,7% | 25,9% | 0,05 |
| DAP3 | 1 | 155656251 | 155658250 | 1 | 32,7% | 24,9% | 0,04 |
| DCUN1D2-AS | 13 | 114121716 | 114123715 | 3 | 33,8% | 31,6% | 0,02 |
| DDX41 | 5 | 176943971 | 176945970 | 7 | 26,9% | 25,7% | 0,04 |
| DENND1C | 19 | 6482069 | 6484068 | 2 | 35,8% | 28,0% | 0,05 |
| DGAT1 | 8 | 145550074 | 145552073 | 12 | 33,0% | 27,1% | 0,05 |
| DHX35 | 20 | 37589442 | 37591441 | 9 | 31,9% | 28,5% | 0,05 |
| DHX36 | 3 | 154041787 | 154043786 | 6 | 38,0% | 32,1% | 0,05 |
| DNAH14 | 1 | 225082464 | 225084463 | 1 | 54,4% | 39,1% | 0,03 |
| DNTTIP2 | 1 | 94344975 | 94346974 | 2 | 45,2% | 33,9% | 0,02 |
| DTNA | 18 | 32071754 | 32073753 | 2 | 35,3% | 29,7% | 0,05 |
| EDDM3A | 14 | 21212551 | 21214550 | 1 | 39,0% | 30,1% | 0,03 |
| EGOT | 3 | 4792775 | 4794774 | 1 | 33,4% | 25,1% | 0,03 |
| EHMT1 | 9 | 140511944 | 140513943 | 3 | 29,3% | 24,4% | 0,04 |
| EIF1AXP1 | 1 | 17012051 | 17014050 | 1 | 42,6% | 30,3% | 0,02 |
| EIF2B5-IT1 | 3 | 184221401 | 184223400 | 1 | 42,4% | 31,7% | 0,05 |
| ENDOU | 12 | 48118851 | 48120850 | 1 | 33,8% | 23,6% | 0,05 |
| EP300 | 22 | 41486290 | 41488289 | 2 | 37,9% | 24,4% | 0,01 |
| ERBB4 | 2 | 213403066 | 213405065 | 7 | 38,9% | 30,3% | 0,04 |
| ERVH48-1 | 21 | 44345257 | 44347256 | 1 | 46,3% | 35,3% | 0,02 |
| ERVK13-1 | 16 | 2722946 | 2724945 | 1 | 52,7% | 36,8% | 0,004 |
| EVX2 | 2 | 176948142 | 176950141 | 4 | 41,3% | 32,7% | 0,05 |
| FAM102B | 1 | 109101211 | 109103210 | 3 | 35,5% | 26,2% | 0,04 |
| FAM170B-AS1 | 10 | 50328384 | 50330383 | 2 | 37,5% | 34,0% | 0,02 |
| FAM205CP | 9 | 34895276 | 34897275 | 1 | 49,0% | 32,7% | 0,02 |
| FAM216B | 13 | 43354186 | 43356185 | 3 | 37,8% | 29,1% | 0,04 |
| FAT3 | 11 | 92083762 | 92085761 | 1 | 60,9% | 37,0% | 0,002 |
| FCN2 | 9 | 137771154 | 137773153 | 1 | 47,1% | 32,8% | 0,05 |
| FEM1AP3 | 6 | 112688147 | 112690146 | 2 | 31,3% | 24,0% | 0,04 |
| FEZF1 | 7 | 121950246 | 121952245 | 2 | 31,0% | 29,3% | 0,04 |
| FNDC9 | 5 | 156772230 | 156774229 | 3 | 37,1% | 29,3% | 0,05 |
| FOXC1 | 6 | 1609181 | 1611180 | 3 | 28,4% | 25,0% | 0,05 |
| GABRA6 | 5 | 160972569 | 160974568 | 4 | 31,6% | 28,7% | 0,03 |
| GAPVD1 | 9 | 128022573 | 128024572 | 2 | 45,2% | 29,2% | 0,01 |
| GAS1 | 9 | 89561605 | 89563604 | 3 | 36,5% | 30,8% | 0,03 |
| GAS5-AS1 | 1 | 173830886 | 173832885 | 1 | 52,4% | 34,7% | 0,02 |
| GHITM | 10 | 85897696 | 85899695 | 3 | 35,9% | 30,4% | 0,02 |
| GLYAT | 11 | 58498948 | 58500947 | 1 | 39,2% | 28,5% | 0,05 |
| GM2A | 5 | 150590211 | 150592210 | 1 | 47,2% | 28,5% | 0,01 |
| GMEB2 | 20 | 62257895 | 62259894 | 6 | 28,4% | 23,5% | 0,02 |
| GPCPD1 | 20 | 5591173 | 5593172 | 2 | 40,5% | 30,3% | 0,03 |
| GPR18 | 13 | 99913499 | 99915498 | 1 | 49,8% | 34,1% | 0,002 |
| GTF2I | 7 | 74070494 | 74072493 | 3 | 31,7% | 23,6% | 0,04 |
| HAR1B | 20 | 61733172 | 61735171 | 2 | 35,3% | 27,3% | 0,05 |
| HAVCR2 | 5 | 156569381 | 156571380 | 2 | 32,1% | 20,2% | 0,01 |
| HBG1 | 11 | 5270623 | 5272622 | 1 | 44,9% | 33,9% | 0,03 |
| HGD | 3 | 120400919 | 120402918 | 1 | 29,5% | 22,1% | 0,03 |
| HIGD1AP15 | 20 | 10313567 | 10315566 | 1 | 35,1% | 24,1% | 0,02 |
| HIST1H2AJ | 6 | 27782108 | 27784107 | 1 | 43,7% | 32,6% | 0,03 |
| HIST1H2BPS3 | 13 | 22057814 | 22059813 | 1 | 31,4% | 22,0% | 0,01 |
| HLA-K | 6 | 29892736 | 29894735 | 1 | 35,0% | 20,9% | 0,01 |
| HMGB2 | 4 | 174255777 | 174257776 | 3 | 37,5% | 29,8% | 0,01 |
| HN1L | 16 | 1726757 | 1728756 | 5 | 32,0% | 26,8% | 0,02 |
| HSPE1P12 | 12 | 10921404 | 10923403 | 1 | 48,2% | 34,4% | 0,05 |
| IFNGR2 | 21 | 34773702 | 34775701 | 3 | 33,9% | 25,7% | 0,04 |
| IFRD1 | 7 | 112061523 | 112063522 | 1 | 48,4% | 33,4% | 0,01 |
| IGHD1-7 | 14 | 106375283 | 106377282 | 1 | 32,0% | 24,1% | 0,05 |
| IGHD6-6 | 14 | 106375787 | 106377786 | 1 | 32,0% | 24,1% | 0,05 |
| IGKV1D-42 | 2 | 90227545 | 90229544 | 1 | 43,0% | 30,5% | 0,01 |
| IGLV2-28 | 22 | 23005443 | 23007442 | 1 | 28,1% | 21,2% | 0,03 |
| IGLV4-69 | 22 | 22383832 | 22385831 | 1 | 40,7% | 28,5% | 0,01 |
| IGLV8OR8-1 | 8 | 48114021 | 48116020 | 1 | 42,7% | 30,4% | 0,03 |
| IL19 | 1 | 206970715 | 206972714 | 3 | 38,2% | 32,6% | 0,05 |
| IL1B | 2 | 113593981 | 113595980 | 1 | 48,9% | 32,1% | 0,01 |
| IL6 | 7 | 22764003 | 22766002 | 1 | 38,4% | 28,0% | 0,01 |
| ILF3 | 19 | 10763437 | 10765436 | 6 | 33,2% | 30,0% | 0,05 |
| IPO7 | 11 | 9404669 | 9406668 | 2 | 36,1% | 28,8% | 0,04 |
| IRAK3 | 12 | 66581159 | 66583158 | 3 | 31,4% | 28,8% | 0,04 |
| IRF1 | 5 | 131825991 | 131827990 | 8 | 29,9% | 24,2% | 0,04 |
| ITK | 5 | 156568444 | 156570443 | 1 | 34,6% | 19,3% | 0,01 |
| KCNH5 | 14 | 63568256 | 63570255 | 1 | 67,6% | 40,8% | 0,004 |
| KDELR1 | 19 | 48894311 | 48896310 | 8 | 32,8% | 29,5% | 0,03 |
| KDM4B | 19 | 4967625 | 4969624 | 1 | 37,8% | 26,1% | 0,01 |
| KIF13A | 6 | 17987355 | 17989354 | 4 | 34,5% | 26,8% | 0,04 |
| KLF11 | 2 | 10181476 | 10183475 | 2 | 30,2% | 26,9% | 0,03 |
| KLHDC10 | 7 | 129708850 | 129710849 | 2 | 48,3% | 31,5% | 0,01 |
| KLK8 | 19 | 51505468 | 51507467 | 1 | 39,0% | 30,5% | 0,03 |
| KNG1 | 3 | 186433565 | 186435564 | 1 | 33,8% | 25,7% | 0,03 |
| KRT24 | 17 | 38859503 | 38861502 | 2 | 36,7% | 27,0% | 0,03 |
| KRTAP20-2 | 21 | 32006055 | 32008054 | 2 | 41,8% | 31,9% | 0,02 |
| KRTAP4-1 | 17 | 39341095 | 39343094 | 1 | 38,5% | 26,2% | 0,01 |
| KRTAP9-9 | 17 | 39410136 | 39412135 | 1 | 39,0% | 26,7% | 0,01 |
| LARP1 | 5 | 154090962 | 154092961 | 2 | 33,2% | 28,5% | 0,02 |
| LBH | 2 | 30452897 | 30454896 | 5 | 25,9% | 24,1% | 0,03 |
| LEMD3 | 12 | 65561851 | 65563850 | 3 | 30,5% | 21,2% | 0,03 |
| LHFP | 13 | 40177166 | 40179165 | 1 | 32,7% | 24,8% | 0,02 |
| LINC-ROR | 18 | 54738851 | 54740850 | 1 | 39,9% | 27,6% | 0,03 |
| LINC00092 | 9 | 98789748 | 98791747 | 2 | 42,9% | 29,4% | 0,03 |
| LINC00237 | 20 | 21086500 | 21088499 | 3 | 35,6% | 29,5% | 0,05 |
| LINC00332 | 13 | 40754446 | 40756445 | 1 | 48,1% | 28,6% | 0,003 |
| LINC00358 | 13 | 62603182 | 62605181 | 1 | 43,5% | 30,5% | 0,01 |
| LINC00401 | 13 | 69883965 | 69885964 | 1 | 36,3% | 24,2% | 0,002 |
| LINC00443 | 13 | 107304764 | 107306763 | 1 | 38,5% | 26,3% | 0,01 |
| LINC00471 | 2 | 232378551 | 232380550 | 1 | 34,8% | 25,6% | 0,04 |
| LINC00520 | 14 | 56262907 | 56264906 | 1 | 55,2% | 35,8% | 0,003 |
| LINC00674 | 17 | 66096549 | 66098548 | 2 | 40,4% | 31,3% | 0,01 |
| LINC01096 | 4 | 13548926 | 13550925 | 4 | 30,8% | 28,1% | 0,04 |
| LONP1 | 19 | 5720084 | 5722083 | 8 | 34,4% | 27,9% | 0,04 |
| LPP-AS1 | 3 | 188285955 | 188287954 | 1 | 39,7% | 27,9% | 0,03 |
| LRRC37A7P | 18 | 29302661 | 29304660 | 1 | 40,7% | 24,1% | 0,001 |
| LSM3P3 | 2 | 85329318 | 85331317 | 2 | 40,3% | 29,7% | 0,02 |
| LTA | 6 | 31538331 | 31540330 | 5 | 31,3% | 28,8% | 0,04 |
| MANBA | 4 | 103681652 | 103683651 | 6 | 29,9% | 24,2% | 0,05 |
| MANSC1 | 12 | 12502976 | 12504975 | 2 | 36,4% | 25,2% | 0,01 |
| MAP3K6 | 1 | 27692884 | 27694883 | 4 | 32,9% | 27,7% | 0,04 |
| MBNL1 | 3 | 151960117 | 151962116 | 1 | 34,4% | 26,9% | 0,04 |
| MFI2 | 3 | 196756188 | 196758187 | 5 | 34,1% | 30,0% | 0,03 |
| MFSD2B | 2 | 24231451 | 24233450 | 7 | 41,7% | 32,0% | 0,05 |
| MIR100 | 11 | 122022517 | 122024516 | 1 | 31,1% | 24,0% | 0,05 |
| MIR1281 | 22 | 41487017 | 41489016 | 3 | 35,0% | 26,2% | 0,04 |
| MIR1289-2 | 5 | 132762899 | 132764898 | 2 | 43,2% | 36,1% | 0,01 |
| MIR1304 | 11 | 93466431 | 93468430 | 1 | 37,2% | 27,9% | 0,03 |
| MIR208A | 14 | 23857382 | 23859381 | 1 | 36,8% | 28,1% | 0,05 |
| MIR3679 | 2 | 134883196 | 134885195 | 2 | 34,9% | 30,0% | 0,03 |
| MIR4262 | 2 | 11976613 | 11978612 | 1 | 54,5% | 38,0% | 0,01 |
| MIR549 | 15 | 81133915 | 81135914 | 1 | 38,9% | 26,3% | 0,01 |
| MIR554 | 1 | 151516772 | 151518771 | 2 | 43,0% | 32,4% | 0,04 |
| MIR5699 | 10 | 687219 | 689218 | 1 | 47,6% | 26,6% | 0,005 |
| MLF2 | 12 | 6876142 | 6878141 | 4 | 40,1% | 34,0% | 0,01 |
| MMP10 | 11 | 102650860 | 102652859 | 1 | 63,9% | 39,9% | 0,004 |
| MPRIP | 17 | 16944359 | 16946358 | 3 | 27,0% | 25,2% | 0,04 |
| MRAP | 21 | 33662624 | 33664623 | 1 | 43,9% | 30,6% | 0,01 |
| MRPL37 | 1 | 54648214 | 54650213 | 1 | 57,9% | 36,0% | 0,01 |
| MSX2P1 | 17 | 56232889 | 56234888 | 1 | 47,3% | 31,1% | 0,001 |
| MTMR6 | 13 | 25861648 | 25863647 | 2 | 46,8% | 30,5% | 0,003 |
| MUCL1 | 12 | 55222803 | 55224802 | 1 | 39,9% | 23,6% | 0,003 |
| MYCT1 | 6 | 153017530 | 153019529 | 1 | 46,8% | 34,6% | 0,02 |
| MYRIP | 3 | 39848905 | 39850904 | 3 | 35,2% | 35,2% | 0,04 |
| NAALADL2-AS1 | 3 | 175493622 | 175495621 | 1 | 46,3% | 33,6% | 0,04 |
| NARFL | 16 | 790830 | 792829 | 6 | 29,2% | 25,3% | 0,05 |
| NBR1 | 17 | 41320998 | 41322997 | 1 | 45,1% | 30,4% | 0,04 |
| NCOA7 | 6 | 126100807 | 126102806 | 2 | 27,4% | 22,1% | 0,03 |
| NDNF | 4 | 121993677 | 121995676 | 2 | 34,0% | 25,9% | 0,05 |
| NDUFB2 | 7 | 140389077 | 140391076 | 2 | 35,6% | 31,4% | 0,03 |
| NETO2 | 16 | 47177409 | 47179408 | 8 | 31,9% | 26,5% | 0,05 |
| NFYB | 12 | 104531568 | 104533567 | 3 | 33,3% | 26,0% | 0,03 |
| NHP2P1 | 10 | 93974589 | 93976588 | 1 | 51,7% | 29,3% | 0,001 |
| NIPAL2 | 8 | 99306122 | 99308121 | 3 | 30,8% | 30,0% | 0,03 |
| NPS | 10 | 129346113 | 129348112 | 1 | 32,1% | 22,1% | 0,005 |
| NPTXR | 22 | 39239488 | 39241487 | 4 | 33,2% | 30,6% | 0,05 |
| NR2E1 | 6 | 108485762 | 108487761 | 6 | 31,2% | 25,8% | 0,02 |
| NR2F1 | 5 | 92917543 | 92919542 | 1 | 39,6% | 26,7% | 0,02 |
| NRG1-IT3 | 8 | 32296762 | 32298761 | 1 | 42,1% | 32,6% | 0,05 |
| NUS1P3 | 13 | 24900849 | 24902848 | 1 | 46,1% | 34,2% | 0,04 |
| NYAP2 | 2 | 226263864 | 226265863 | 1 | 52,6% | 29,1% | 0,001 |
| OCM2 | 7 | 97619982 | 97621981 | 1 | 35,1% | 26,6% | 0,03 |
| OLA1P3 | 12 | 56265887 | 56267886 | 1 | 40,9% | 25,5% | 0,01 |
| OR10A2 | 11 | 6889424 | 6891423 | 1 | 36,8% | 28,1% | 0,04 |
| OR10H4 | 19 | 16058318 | 16060317 | 2 | 30,8% | 27,9% | 0,01 |
| OR10K2 | 1 | 158390157 | 158392156 | 1 | 36,5% | 26,5% | 0,01 |
| OR1A2 | 17 | 3099313 | 3101312 | 2 | 37,9% | 28,0% | 0,02 |
| OR1G1 | 17 | 3030376 | 3032375 | 1 | 32,5% | 21,5% | 0,01 |
| OR1J2 | 9 | 125271581 | 125273580 | 1 | 53,8% | 36,8% | 0,01 |
| OR1Q1 | 9 | 125375448 | 125377447 | 1 | 46,7% | 36,3% | 0,05 |
| OR2T6 | 1 | 248549410 | 248551409 | 1 | 32,4% | 23,8% | 0,01 |
| OR4D1 | 17 | 56230994 | 56232993 | 3 | 38,6% | 29,5% | 0,03 |
| OR4D9 | 11 | 59280886 | 59282885 | 1 | 41,1% | 30,7% | 0,04 |
| OR5B12 | 11 | 58207147 | 58209146 | 1 | 30,9% | 24,1% | 0,05 |
| OR6S1 | 14 | 21109351 | 21111350 | 2 | 37,8% | 33,0% | 0,02 |
| OR7A10 | 19 | 14952190 | 14954189 | 1 | 33,4% | 26,1% | 0,05 |
| OR7G1 | 19 | 9225940 | 9227939 | 1 | 46,9% | 36,0% | 0,03 |
| OR8D4 | 11 | 123775609 | 123777608 | 1 | 42,9% | 33,1% | 0,05 |
| P2RY14 | 3 | 150995756 | 150997755 | 1 | 35,2% | 25,2% | 0,04 |
| PA2G4P4 | 3 | 156528022 | 156530021 | 1 | 35,3% | 27,4% | 0,04 |
| PCDHB11 | 5 | 140577683 | 140579682 | 1 | 35,9% | 28,4% | 0,05 |
| PCDHGA7 | 5 | 140760967 | 140762966 | 2 | 38,6% | 31,9% | 0,04 |
| PCDHGC3 | 5 | 140854080 | 140856079 | 3 | 33,2% | 25,5% | 0,05 |
| PDE6H | 12 | 15124456 | 15126455 | 1 | 36,5% | 26,5% | 0,02 |
| PEX13 | 2 | 61242860 | 61244859 | 2 | 42,6% | 32,9% | 0,05 |
| PHBP1 | 6 | 150362182 | 150364181 | 2 | 33,6% | 32,5% | 0,04 |
| PHC1 | 12 | 9064992 | 9066991 | 2 | 35,4% | 29,2% | 0,04 |
| PHF10 | 6 | 170123652 | 170125651 | 2 | 28,2% | 28,1% | 0,01 |
| PIK3C2G | 12 | 18399048 | 18401047 | 1 | 32,5% | 25,2% | 0,03 |
| PIK3C3 | 18 | 39533671 | 39535670 | 3 | 39,0% | 31,2% | 0,01 |
| PKP2 | 12 | 33049275 | 33051274 | 3 | 39,6% | 26,7% | 0,01 |
| PLCG2 | 16 | 81771202 | 81773201 | 1 | 33,2% | 23,8% | 0,04 |
| PLCL1 | 2 | 198667926 | 198669925 | 5 | 36,1% | 28,4% | 0,05 |
| PLEKHG7 | 12 | 93113781 | 93115780 | 1 | 46,8% | 33,4% | 0,02 |
| PNLIPRP1 | 10 | 118348397 | 118350396 | 1 | 53,3% | 31,4% | 0,00002 |
| PNMA1 | 14 | 74180629 | 74182628 | 1 | 32,9% | 22,4% | 0,01 |
| PNPLA2 | 11 | 817402 | 819401 | 4 | 37,6% | 28,9% | 0,02 |
| POM121L2 | 6 | 27279450 | 27281449 | 4 | 34,0% | 27,9% | 0,05 |
| POMGNT2 | 3 | 43147069 | 43149068 | 3 | 37,9% | 29,2% | 0,05 |
| POU5F1B | 8 | 128425035 | 128427034 | 1 | 60,7% | 44,6% | 0,03 |
| PPARGC1A | 4 | 23905213 | 23907212 | 1 | 42,7% | 27,3% | 0,02 |
| PPIL6 | 6 | 109761875 | 109763874 | 7 | 30,1% | 23,5% | 0,05 |
| PPP1R1B | 17 | 37781493 | 37783492 | 6 | 30,8% | 28,9% | 0,03 |
| PPP1R1C | 2 | 182817468 | 182819467 | 1 | 45,5% | 34,5% | 0,01 |
| PRKACB | 1 | 84542245 | 84544244 | 3 | 33,5% | 24,6% | 0,03 |
| PRKAG1 | 12 | 49412481 | 49414480 | 5 | 29,7% | 28,4% | 0,04 |
| PRKAR1A | 17 | 66506421 | 66508420 | 5 | 30,1% | 27,4% | 0,04 |
| PRKAR2B | 7 | 106683594 | 106685593 | 4 | 31,5% | 23,9% | 0,03 |
| PROS1 | 3 | 93692411 | 93694410 | 3 | 35,6% | 29,5% | 0,05 |
| PRR18 | 6 | 166721437 | 166723436 | 1 | 36,7% | 26,3% | 0,05 |
| PRRX2 | 9 | 132426420 | 132428419 | 1 | 38,4% | 22,1% | 0,0004 |
| PRSS3 | 9 | 33749015 | 33751014 | 2 | 43,4% | 28,9% | 0,03 |
| PTGS2 | 1 | 186649060 | 186651059 | 2 | 32,3% | 23,8% | 0,02 |
| PTP4A2P1 | 17 | 40684050 | 40686049 | 1 | 65,0% | 42,8% | 0,02 |
| PTPRB | 12 | 71030721 | 71032720 | 2 | 45,3% | 34,7% | 0,05 |
| PTPRH | 19 | 55720375 | 55722374 | 3 | 39,6% | 34,6% | 0,01 |
| PTPRM | 18 | 7565280 | 7567279 | 3 | 33,3% | 23,5% | 0,03 |
| PURA | 5 | 139485862 | 139487861 | 1 | 26,5% | 17,9% | 0,04 |
| RAB11B-AS1 | 19 | 8455070 | 8457069 | 3 | 35,7% | 26,3% | 0,05 |
| REG4 | 1 | 120353784 | 120355783 | 1 | 35,8% | 26,0% | 0,02 |
| RGS17P1 | 13 | 41566968 | 41568967 | 1 | 34,7% | 25,4% | 0,05 |
| RHOH | 4 | 40191173 | 40193172 | 2 | 35,3% | 26,4% | 0,03 |
| RHPN2 | 19 | 33555295 | 33557294 | 3 | 29,3% | 28,6% | 0,01 |
| RMND5A | 2 | 86945796 | 86947795 | 2 | 47,7% | 26,0% | 0,002 |
| RMRPP4 | 10 | 52344964 | 52346963 | 1 | 36,9% | 27,2% | 0,03 |
| RMRPP5 | 9 | 24903967 | 24905966 | 1 | 47,7% | 34,0% | 0,002 |
| RN7SKP140 | 20 | 21405180 | 21407179 | 1 | 41,4% | 31,1% | 0,02 |
| RN7SKP257 | 14 | 37062853 | 37064852 | 2 | 41,1% | 28,2% | 0,01 |
| RN7SKP34 | 5 | 86398164 | 86400163 | 1 | 50,8% | 32,7% | 0,01 |
| RN7SKP43 | 2 | 218114779 | 218116778 | 1 | 41,5% | 30,3% | 0,01 |
| RN7SKP48 | 4 | 86020149 | 86022148 | 1 | 43,1% | 30,2% | 0,04 |
| RN7SL102P | 17 | 36777234 | 36779233 | 1 | 35,7% | 27,1% | 0,04 |
| RNA5SP187 | 5 | 87569694 | 87571693 | 1 | 40,2% | 28,2% | 0,01 |
| RNA5SP405 | 16 | 26038733 | 26040732 | 1 | 36,2% | 24,4% | 0,04 |
| RNA5SP471 | 19 | 32145868 | 32147867 | 1 | 35,5% | 27,0% | 0,04 |
| RNA5SP85 | 2 | 11701406 | 11703405 | 1 | 31,5% | 24,3% | 0,02 |
| RNASE12 | 14 | 21058483 | 21060482 | 1 | 52,9% | 34,3% | 0,003 |
| RNF141 | 11 | 10562278 | 10564277 | 6 | 30,7% | 26,1% | 0,05 |
| RNU1-21P | 11 | 123633135 | 123635134 | 1 | 36,4% | 25,8% | 0,01 |
| RNU1-22P | 16 | 3134644 | 3136643 | 1 | 32,3% | 24,4% | 0,03 |
| RNU1-23P | 20 | 24216391 | 24218390 | 1 | 34,3% | 23,0% | 0,02 |
| RNU1-69P | 12 | 56266962 | 56268961 | 1 | 40,9% | 25,5% | 0,01 |
| RNU2-47P | 9 | 12299952 | 12301951 | 1 | 38,3% | 25,5% | 0,004 |
| RNU2-6P | 13 | 46948226 | 46950225 | 1 | 46,0% | 28,3% | 0,003 |
| RNU2-8P | 6 | 121901168 | 121903167 | 1 | 39,5% | 27,5% | 0,002 |
| RNU4ATAC6P | 10 | 28940059 | 28942058 | 1 | 38,2% | 26,8% | 0,01 |
| RNU5E-1 | 1 | 11966709 | 11968708 | 1 | 46,8% | 33,2% | 0,01 |
| RNU5F-1 | 1 | 45187075 | 45189074 | 1 | 35,8% | 26,9% | 0,01 |
| RNU6-1093P | 12 | 50649628 | 50651627 | 1 | 30,0% | 18,8% | 0,004 |
| RNU6-1141P | 10 | 21950171 | 21952170 | 2 | 32,7% | 26,0% | 0,05 |
| RNU6-1156P | 11 | 125443115 | 125445114 | 1 | 33,9% | 24,0% | 0,05 |
| RNU6-1188P | 12 | 116518875 | 116520874 | 1 | 37,1% | 28,8% | 0,05 |
| RNU6-270P | 10 | 29049233 | 29051232 | 2 | 26,5% | 24,9% | 0,01 |
| RNU6-319P | 9 | 15431505 | 15433504 | 1 | 35,3% | 27,2% | 0,04 |
| RNU6-38P | 13 | 75682224 | 75684223 | 1 | 46,1% | 29,6% | 0,004 |
| RNU6-442P | 8 | 126911695 | 126913694 | 1 | 53,7% | 37,1% | 0,01 |
| RNU6-502P | 6 | 26519187 | 26521186 | 1 | 43,3% | 31,4% | 0,01 |
| RNU6-507P | 3 | 149496396 | 149498395 | 1 | 38,7% | 27,3% | 0,05 |
| RNU6-525P | 5 | 179870009 | 179872008 | 1 | 42,7% | 31,2% | 0,02 |
| RNU6-547P | 3 | 172419414 | 172421413 | 2 | 55,5% | 37,2% | 0,003 |
| RNU6-63P | 13 | 28060775 | 28062774 | 1 | 38,2% | 29,2% | 0,04 |
| RNU6-723P | 1 | 48808381 | 48810380 | 1 | 51,1% | 37,3% | 0,03 |
| RNU6-724P | 5 | 68826045 | 68828044 | 1 | 33,0% | 23,6% | 0,03 |
| RNU6-768P | 12 | 101592656 | 101594655 | 1 | 40,5% | 27,3% | 0,001 |
| RNU6-76P | 13 | 19452694 | 19454693 | 1 | 56,4% | 38,8% | 0,01 |
| RNU6-794P | 10 | 35519823 | 35521822 | 1 | 41,5% | 25,5% | 0,0002 |
| RNU6-802P | 14 | 24820864 | 24822863 | 2 | 35,5% | 27,2% | 0,05 |
| RNU6-824P | 6 | 155145914 | 155147913 | 2 | 38,1% | 28,8% | 0,02 |
| RNU6-853P | 4 | 169805006 | 169807005 | 1 | 34,0% | 26,1% | 0,05 |
| RNU6-865P | 3 | 100669971 | 100671970 | 1 | 30,9% | 21,4% | 0,03 |
| RNU6-914P | 8 | 99204042 | 99206041 | 1 | 38,4% | 28,1% | 0,01 |
| RNU6-986P | 2 | 27697963 | 27699962 | 1 | 37,9% | 29,7% | 0,03 |
| RNU6ATAC6P | 3 | 87957870 | 87959869 | 1 | 40,0% | 29,1% | 0,03 |
| RNU7-126P | 4 | 25599748 | 25601747 | 1 | 39,0% | 24,9% | 0,01 |
| RNU7-181P | 8 | 131016348 | 131018347 | 1 | 44,3% | 33,0% | 0,02 |
| RNU7-192P | 4 | 189637380 | 189639379 | 2 | 41,9% | 32,0% | 0,03 |
| RNY4 | 7 | 148658907 | 148660906 | 2 | 35,3% | 25,1% | 0,04 |
| RPL23AP1 | 6 | 29694417 | 29696416 | 1 | 43,2% | 30,2% | 0,01 |
| RPL34P6 | 1 | 200831436 | 200833435 | 1 | 34,9% | 23,9% | 0,02 |
| RPL41 | 12 | 56508870 | 56510869 | 4 | 32,5% | 27,5% | 0,05 |
| RPL7P49 | 17 | 74426707 | 74428706 | 1 | 37,6% | 29,2% | 0,05 |
| RPP38 | 10 | 15137679 | 15139678 | 5 | 33,4% | 23,4% | 0,03 |
| RPS14 | 5 | 149828820 | 149830819 | 7 | 32,7% | 29,2% | 0,04 |
| RPS15AP10 | 1 | 46111370 | 46113369 | 1 | 31,6% | 23,2% | 0,03 |
| RWDD4 | 4 | 184579879 | 184581878 | 3 | 38,2% | 30,9% | 0,03 |
| SCAPER | 15 | 77197286 | 77199285 | 4 | 27,6% | 25,4% | 0,04 |
| SCARNA6 | 2 | 234195822 | 234197821 | 1 | 39,2% | 31,6% | 0,05 |
| SCD | 10 | 102105381 | 102107380 | 6 | 28,0% | 26,8% | 0,05 |
| SCIMP | 17 | 5137656 | 5139655 | 5 | 40,0% | 34,8% | 0,05 |
| SDAD1P1 | 8 | 26239970 | 26241969 | 7 | 29,2% | 26,3% | 0,04 |
| SDR42E1 | 16 | 82044594 | 82046593 | 4 | 36,6% | 28,1% | 0,04 |
| SERTAD4-AS1 | 1 | 210406893 | 210408892 | 2 | 29,7% | 23,1% | 0,04 |
| SHANK2 | 11 | 70963124 | 70965123 | 3 | 33,2% | 24,2% | 0,02 |
| SIK3 | 11 | 116968654 | 116970653 | 5 | 31,2% | 27,6% | 0,03 |
| SLC17A1 | 6 | 25831788 | 25833787 | 2 | 42,5% | 33,2% | 0,05 |
| SLC17A4 | 6 | 25753427 | 25755426 | 1 | 39,4% | 29,5% | 0,03 |
| SLC18A3 | 10 | 50816847 | 50818846 | 6 | 31,1% | 29,2% | 0,05 |
| SLC26A3 | 7 | 107443171 | 107445170 | 1 | 32,9% | 20,4% | 0,02 |
| SLC36A2 | 5 | 150726652 | 150728651 | 1 | 42,6% | 30,6% | 0,01 |
| SLC6A7 | 5 | 149568020 | 149570019 | 5 | 33,6% | 28,9% | 0,05 |
| SMPD2 | 6 | 109760466 | 109762465 | 7 | 29,7% | 23,6% | 0,05 |
| SMPDL3B | 1 | 28260004 | 28262003 | 2 | 32,3% | 24,6% | 0,04 |
| SNAPC5 | 15 | 66789652 | 66791651 | 1 | 32,8% | 25,7% | 0,05 |
| SNORA18 | 11 | 93466264 | 93468263 | 1 | 37,2% | 27,9% | 0,03 |
| SNORD114-3 | 14 | 101418186 | 101420185 | 2 | 40,8% | 32,2% | 0,03 |
| SNORD114-4 | 14 | 101419211 | 101421210 | 2 | 40,8% | 32,2% | 0,03 |
| SNORD14A | 11 | 17095792 | 17097791 | 1 | 37,0% | 27,5% | 0,05 |
| SNORD5 | 11 | 93465967 | 93467966 | 1 | 37,2% | 27,9% | 0,03 |
| SNRNP48 | 6 | 7588932 | 7590931 | 4 | 35,7% | 27,9% | 0,03 |
| SPRY2 | 13 | 80914587 | 80916586 | 7 | 28,0% | 24,7% | 0,05 |
| SRD5A3P1 | 11 | 59664158 | 59666157 | 1 | 74,4% | 48,0% | 0,01 |
| SSBP1 | 7 | 141436621 | 141438620 | 5 | 35,1% | 29,1% | 0,04 |
| SSBP4 | 19 | 18528174 | 18530173 | 4 | 38,7% | 35,3% | 0,05 |
| SSR3 | 3 | 156272474 | 156274473 | 7 | 30,4% | 27,8% | 0,05 |
| SSU72 | 1 | 1509750 | 1511749 | 8 | 25,9% | 24,4% | 0,02 |
| ST13P4 | 13 | 50744725 | 50746724 | 1 | 64,2% | 46,4% | 0,05 |
| STC2 | 5 | 172756007 | 172758006 | 2 | 27,0% | 24,3% | 0,04 |
| STEAP1B | 7 | 22672045 | 22674044 | 1 | 44,8% | 34,4% | 0,02 |
| STMN4 | 8 | 27115438 | 27117437 | 2 | 31,0% | 30,8% | 0,05 |
| STOML2 | 9 | 35102655 | 35104654 | 1 | 37,0% | 28,2% | 0,04 |
| STT3B | 3 | 31572630 | 31574629 | 4 | 31,1% | 23,8% | 0,05 |
| TANC2 | 17 | 61085417 | 61087416 | 1 | 29,1% | 21,4% | 0,05 |
| TARSL2 | 15 | 102264308 | 102266307 | 3 | 37,6% | 35,4% | 0,04 |
| TAT | 16 | 71610534 | 71612533 | 1 | 43,7% | 29,4% | 0,003 |
| TATDN3 | 1 | 212963670 | 212965669 | 2 | 40,1% | 28,7% | 0,04 |
| TBC1D20 | 20 | 442698 | 444697 | 4 | 29,9% | 28,0% | 0,05 |
| TCEB2P4 | 10 | 34777307 | 34779306 | 1 | 37,8% | 29,9% | 0,04 |
| TECR | 19 | 14626397 | 14628396 | 1 | 30,0% | 21,2% | 0,02 |
| TECRP1 | 4 | 87870777 | 87872776 | 3 | 41,1% | 36,0% | 0,01 |
| TEX10 | 9 | 103114722 | 103116721 | 4 | 32,5% | 24,8% | 0,01 |
| TFEC | 7 | 115799451 | 115801450 | 1 | 37,6% | 27,3% | 0,01 |
| TIGD4 | 4 | 153700417 | 153702416 | 5 | 28,5% | 28,4% | 0,04 |
| TIMD4 | 5 | 156389767 | 156391766 | 1 | 40,9% | 31,8% | 0,03 |
| TIPARP | 3 | 156389524 | 156391523 | 1 | 50,9% | 36,1% | 0,002 |
| TLR3 | 4 | 186988806 | 186990805 | 2 | 33,8% | 31,4% | 0,03 |
| TMA16 | 4 | 164414094 | 164416093 | 5 | 32,5% | 23,9% | 0,05 |
| TMEM244 | 6 | 130182193 | 130184192 | 1 | 39,1% | 26,5% | 0,01 |
| TMEM259 | 19 | 1020618 | 1022617 | 2 | 30,4% | 20,8% | 0,01 |
| TMEM45A | 3 | 100209963 | 100211962 | 3 | 34,6% | 24,2% | 0,003 |
| TMEM5 | 12 | 64172083 | 64174082 | 4 | 35,3% | 27,5% | 0,05 |
| TMEM50B | 21 | 34851819 | 34853818 | 5 | 34,8% | 30,3% | 0,02 |
| TMEM78 | 1 | 229383883 | 229385882 | 1 | 35,9% | 27,8% | 0,05 |
| TMEM81 | 1 | 205053146 | 205055145 | 1 | 51,4% | 37,5% | 0,02 |
| TMPRSS11F | 4 | 68995099 | 68997098 | 1 | 37,3% | 27,2% | 0,03 |
| TMTC2 | 12 | 83079159 | 83081158 | 3 | 34,8% | 27,5% | 0,04 |
| TNN | 1 | 175035494 | 175037493 | 2 | 33,4% | 26,7% | 0,04 |
| TOM1L1 | 17 | 52975248 | 52977247 | 1 | 76,8% | 48,1% | 0,02 |
| TOMM40 | 19 | 45392326 | 45394325 | 5 | 38,6% | 29,9% | 0,02 |
| TRAJ34 | 14 | 22975151 | 22977150 | 1 | 33,4% | 23,8% | 0,03 |
| TRAPPC2P2 | 8 | 72360955 | 72362954 | 1 | 39,9% | 28,7% | 0,04 |
| TRAV19 | 14 | 22474251 | 22476250 | 1 | 38,9% | 29,1% | 0,03 |
| TRIM26BP | 6 | 30204578 | 30206577 | 1 | 37,0% | 24,5% | 0,01 |
| TRIM72 | 16 | 31223842 | 31225841 | 2 | 32,5% | 28,4% | 0,03 |
| TRIP11 | 14 | 92506741 | 92508740 | 1 | 41,1% | 29,8% | 0,03 |
| TRNT1 | 3 | 3167100 | 3169099 | 2 | 30,3% | 22,9% | 0,03 |
| TRPC1 | 3 | 142441416 | 142443415 | 6 | 33,2% | 31,2% | 0,04 |
| TTC4 | 1 | 55179995 | 55181994 | 2 | 38,1% | 32,0% | 0,05 |
| TWISTNB | 7 | 19748211 | 19750210 | 3 | 32,9% | 24,7% | 0,02 |
| TXNL1 | 18 | 54318332 | 54320331 | 4 | 31,9% | 26,0% | 0,05 |
| TYMS | 18 | 656104 | 658103 | 4 | 26,6% | 23,8% | 0,05 |
| UBA5 | 3 | 132371790 | 132373789 | 1 | 40,3% | 29,9% | 0,02 |
| UBE2SP2 | 17 | 18579074 | 18581073 | 1 | 41,1% | 32,9% | 0,05 |
| UBE2T | 1 | 202310609 | 202312608 | 2 | 31,2% | 23,9% | 0,03 |
| UBN2 | 7 | 138913602 | 138915601 | 1 | 42,3% | 31,9% | 0,03 |
| UCP1 | 4 | 141489460 | 141491459 | 5 | 33,8% | 28,6% | 0,05 |
| UIMC1 | 5 | 176449135 | 176451134 | 8 | 35,1% | 28,8% | 0,05 |
| USP34 | 2 | 61697405 | 61699404 | 5 | 26,3% | 25,7% | 0,05 |
| VAV3 | 1 | 108507267 | 108509266 | 6 | 31,5% | 24,0% | 0,04 |
| VAV3-AS1 | 1 | 108505565 | 108507564 | 4 | 32,2% | 26,0% | 0,04 |
| VIM | 10 | 17268758 | 17270757 | 4 | 32,7% | 31,1% | 0,05 |
| VPS11 | 11 | 118936903 | 118938902 | 5 | 33,2% | 27,9% | 0,05 |
| WBP2 | 17 | 73852089 | 73854088 | 1 | 30,1% | 22,1% | 0,02 |
| WBP2P1 | 18 | 22596669 | 22598668 | 1 | 53,1% | 38,4% | 0,04 |
| WEE2-AS1 | 7 | 141437647 | 141439646 | 5 | 35,1% | 29,1% | 0,04 |
| WTIP | 19 | 34970374 | 34972373 | 2 | 31,4% | 29,8% | 0,02 |
| XRCC1 | 19 | 44084126 | 44086125 | 2 | 35,8% | 26,6% | 0,02 |
| YPEL4 | 11 | 57416918 | 57418917 | 1 | 47,9% | 31,5% | 0,01 |
| YY1 | 14 | 100703135 | 100705134 | 5 | 29,0% | 25,4% | 0,05 |
| ZC3H8 | 2 | 113012214 | 113014213 | 3 | 25,3% | 25,0% | 0,05 |
| ZFP91 | 11 | 58345084 | 58347083 | 1 | 31,2% | 23,3% | 0,04 |
| ZFP91-CNTF | 11 | 58345145 | 58347144 | 1 | 31,2% | 23,3% | 0,04 |
| ZNF205-AS1 | 16 | 3165100 | 3167099 | 1 | 37,6% | 29,3% | 0,05 |
| ZNF24 | 18 | 32924947 | 32926946 | 1 | 41,2% | 30,4% | 0,01 |
| ZNF410 | 14 | 74351820 | 74353819 | 3 | 39,7% | 34,6% | 0,02 |
| ZNF430 | 19 | 21201926 | 21203925 | 1 | 31,8% | 22,0% | 0,004 |
| ZNF706 | 8 | 102217922 | 102219921 | 8 | 27,0% | 23,2% | 0,04 |
| ZNF815P | 7 | 5861291 | 5863290 | 2 | 37,0% | 24,7% | 0,02 |
| ZSWIM5 | 1 | 45771382 | 45773381 | 1 | 38,6% | 30,3% | 0,03 |
